# Bistability: Requirements on Cell-Volume, Protein Diffusion, and Thermodynamics

**DOI:** 10.1371/journal.pone.0121681

**Published:** 2015-04-15

**Authors:** Robert G. Endres

**Affiliations:** Department of Life Sciences & Centre for Integrative Systems Biology and Bioinformatics, London, United Kingdom; Tata Institute of Fundamental Research, INDIA

## Abstract

Bistability is considered wide-spread among bacteria and eukaryotic cells, useful e.g. for enzyme induction, bet hedging, and epigenetic switching. However, this phenomenon has mostly been described with deterministic dynamic or well-mixed stochastic models. Here, we map known biological bistable systems onto the well-characterized biochemical Schlögl model, using analytical calculations and stochastic spatiotemporal simulations. In addition to network architecture and strong thermodynamic driving away from equilibrium, we show that bistability requires fine-tuning towards small cell volumes (or compartments) and fast protein diffusion (well mixing). Bistability is thus fragile and hence may be restricted to small bacteria and eukaryotic nuclei, with switching triggered by volume changes during the cell cycle. For large volumes, single cells generally loose their ability for bistable switching and instead undergo a first-order phase transition.

## Introduction

Isogenetic cell populations show remarkable heterogeneity due to unavoidable molecular noise—bacteria are either induced or uninduced to produce enzymes for utilizing a particular sugar [1], or enter cellular programs such as competence during starvation in a reversible, switch-like manner [2]. In higher organisms examples of bistability are maturation in developing oocytes in *Xenopus* frog embryos [3], Hedgehog signaling in stem cells [4], and phosphorylation-dephosphorylation cycles, e.g. as occurring in mitogen-activated protein kinase (MAPK) cascades [5]. Bistable pathway designs have also been explored in synthetic biology [6–10]. In analogy with physical bistable systems such as ferromagnets, biological cellular systems can indeed exhibit hysteresis, indicative of a system’s memory of past conditions [1, 6–8]. Functional advantages of bistability include bet-hedging strategies, decision-making, specialization, and mechanisms for epigenetic inheritance, all increasing the species’ fitness [11, 12]. However, these phenomena have mostly been described with deterministic dynamic models or well-mixed stochastic models. It is unclear if bistability predicted by the deterministic model always corresponds to a bimodal probability distribution in the stochastic approach [13]. Furthermore, the influence of slow protein diffusion and localization inside the cytoplasm (bacteria) or nucleus (eukaryotes) is often neglected. Whether bistability is robust to such perturbations is unclear.

The question of the role of reaction volume in well-mixed bistable chemical reactions has a long history, e.g. [13–17]. Particularly noteworthy, Keizer’s paradox says that microscopic and macroscopic descriptions can yield different predictions [13]. In the macroscopic description the steady-state (*t* → ∞) is considered after taking the infinite volume limit (*V* → ∞), while in the microscopic description the opposite order of limits is taken. Since the orders are not always interchangeable [18–20], unexpected results can occur. For instance, in the logistic growth equation species extinction occurs in the microscopic description, while the macroscopic description predicts a stable finite steady-state population [21]. As a consequence, in bistable systems it is generally not possible to derive Fokker-Planck or Langevin equations that produces a behavior in accordance with the master equation [13, 22]. Derived potentials determining the weights of the states are incorrect. More sophisticated approximations (or modified Fokker-Planck approaches) are required to capture rare large fluctuations [22, 23], which ultimately determine the switching between states. However, the biological implications of these issues on cell-fate decisions have been rather unexplored, with some exceptions [24, 25].

Furthermore, two recent papers address the effects of diffusion on bistability and switching of states. Zuk *et al.* considered a one-dimensional (1D) and a hexagonal 2D lattice model [16], while Tanase-Nicola and Lubensky considered an 1D *M*-compartment model with hopping between the *M* compartments to represent diffusion [17]. Based on their results, when the system size is small such systems are effectively well-mixed and transitions are driven solely by stochastic fluctuations in line with the well-mixed master equation. However, when the system is spatially extended the more stable state spreads out in space and overtakes the more unstable state by the mechanism of traveling waves. Interestingly, in presence of diffusion the stability of steady states in the extended system is determined by the deterministic (mean-field) potential, which also describes the speed of the traveling waves. However, it is unclear if these results also hold in 3D, for small volumes comparable to nuclei and cytoplasms in cells, and using more realistic particle-based approaches.

Living cells are open molecular systems, characterized by chemical driving forces and free-energy dissipation [26, 27]. Here, we map known biological bistable systems onto the well-characterized non-equilibrium biochemical Schlögl model [14] (recently reviewed in [13]), allowing us to obtain analytical results for the well-mixed case. For slow diffusion we use stochastic spatio-temporal simulations. In addition to network architecture and strong thermodynamic driving away from equilibrium, we show that bistability requires fine-tuning towards small cell volumes (or compartments) and fast protein diffusion (well mixing). Bistability is thus fragile and hence may provide upper limits on cell or nuclear sizes. For increasing volume, a separation of time scales occurs and switching does not only become infinitesimally (exponentially) rare but the weights of the states shift as well. Although states do not disappear per se, weights can disappear, leading effectively to monostability. Hence, single cells loose their ability for reversible bistable switching and instead undergo a first-order phase transition similar to mesoscopic physical systems. Strict cell and nuclear size control may provide a protective molecular environment for bistability. Indeed, our analysis of previously published time-lapse movies of bacteria indicates that volume changes during cell growth and division may function as triggers for switching.

## Results

### Mapping of bistable systems onto Schlögl model

**Fig 1:**
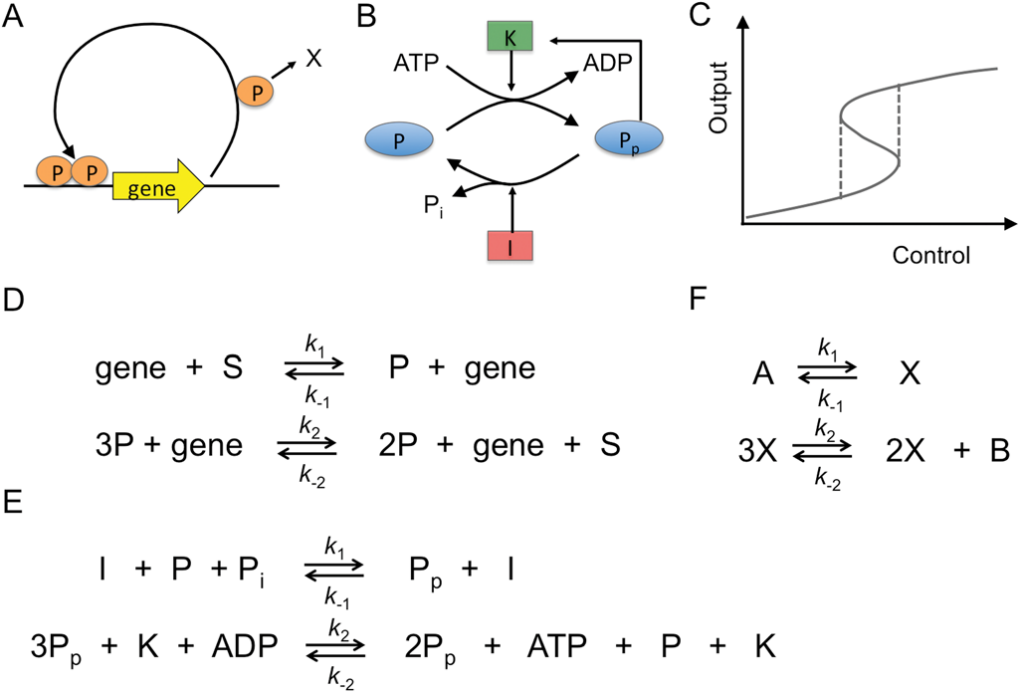
Mapping of bistable systems onto Schlögl model. (A) Self-activating gene with cooperativity. (B) Phosphorylation-dephosphorylation cycle. (C) Schematic bifurcation diagram with bistable regime indicated by vertical dashed lines. (D, E) Chemical reactions corresponding to (A) and (B), respectively. (D) *S* is substrate (nucleotides for mRNA and amino acids for protein etc.) and *P* is protein product. (E) Quantities *I*, *K*, *P* (*P*_*p*_), and *P*_*i*_ are the inhibitor, kinase, (phosphorylated) protein, and inorganic phosphate, respectively. (F) Chemical reactions of Schlögl model with concentrations *A* and *B* adjustable parameters. For mapping reactions in (D) onto reactions in (F) gene species needs to be absorbed into rate constants, and *S* and *P* identified with *A*/*B* and *X*, respectively. For mapping (E) onto (F) *I*, *K*, ADP, and *P* need to be absorbed into rate constants, and *P*_*p*_ identified with *X*, *P*_*i*_ with *A*, and ATP with *B*.

Bistability is driven by high-energy fuel molecules such as ATP and sources of precursor molecules [28]. Here, we choose the self-activating gene, whose protein product binds its own promoter region to cooperatively activate its own transcription as a dimer (see Fig 1A, mRNA is not explicitly modeled here). In addition to ATP required for charging synthetase with amino acids and tRNAs, high-energy molecules involved are nucleotide triphosphates during transcription and GTP during translation [29]. Additionally, we consider the phosphorylation-dephosphorylation cycle with the phosphorylation reaction catalyzed by kinase *K* and the dephosphorylation reaction catalyzed by phosphatase (inhibitor) *I* (Fig 1B) [28]. Also in this case there is positive feedback from product *P*_*p*_ to its production. The mean-field equations, given by ordinary differential equations (ODEs), describe the temporal dynamics of the average protein concentrations, valid in the limit of large volume (and hence large protein copy numbers). At steady state, when all time-derivatives are zero, the equations produce a bistable bifurcation diagram for suitable parameter regimes (see Fig 1C for a schematic). The control parameter (*x*-axis) is a parameter of the model, e.g. a rate constant, and the output (*y*-axis) is the target protein or the phosphorylated protein concentration, respectively.

The chemical reactions for the self-activating gene and the phosphorylation-dephosphorylation cycle with their rate constants are shown in Figs 1D and 1E, respectively. In Fig 1D the first equation indicates basal expression, while the second indicates cooperative self-activation with *S* the substrate (e.g. nucleotides for mRNA and amino acids for protein production). In Fig 1E, the substrate is given by ATP, which is converted to ADP. *P*_*i*_ is inorganic phosphate produced during dephosphorylation, which again is converted into ATP by the cell. Note, while the reverse reactions from protein to substrate *S* (Fig 1D) and protein phosphorylation by *I* or protein dephosphorylation by *K* are extremely unlikely, they technically are nonzero and need to be included for thermodynamic consistency. Importantly, the individual reactions can be mapped onto the well-characterized single-species Schlögl model, in which molecular concentrations *A* and *B* are fixed to drive the reactions out of equilibrium.

The mapping is justified based on the one-to-one correspondence of the molecular reactions (see Fig 1F). For this, however, to work the biological examples would need to be implemented by mass-action kinetics instead of more realistic enzyme-driven kinetics. For instance, the self-activating gene might be implemented by *dp*/*dt* = *a* + *bp* ^2^/(*K* ^2^ + *p* ^2^) − *τ* ^−1^ *p* to describe cooperative self-induction with Hill coefficient 2, protein life time *τ*, and additional parameters *a*, *b* and *K*. While for the self-activating gene *p*-dependent production is to lowest order ∼ *p* ^2^ similar to the Schlögl model (with rate constant *k*_−2_), its reverse rate is assumed to be zero as the forward rate is highly driven by several enzymatic steps. In contrast, in the Schlögl model the reverse rate is assumed to be non-zero (with rate constant *k*_+2_). Similarly, while degradation in the Schlögl model has a reverse rate (“accidental” production from constituents via rate constant *k*_+1_) degradation in gene regulation is either implemented by active degradation or dilution during cell division, both of which have negligible reverse rates. As a result, the macroscopic equation provided by the Schlögl model is a third-order polynomial with rather large reverse reactions due to the absence of enzymatically driven reactions.

### Macroscopic perspective

**Fig 2:**
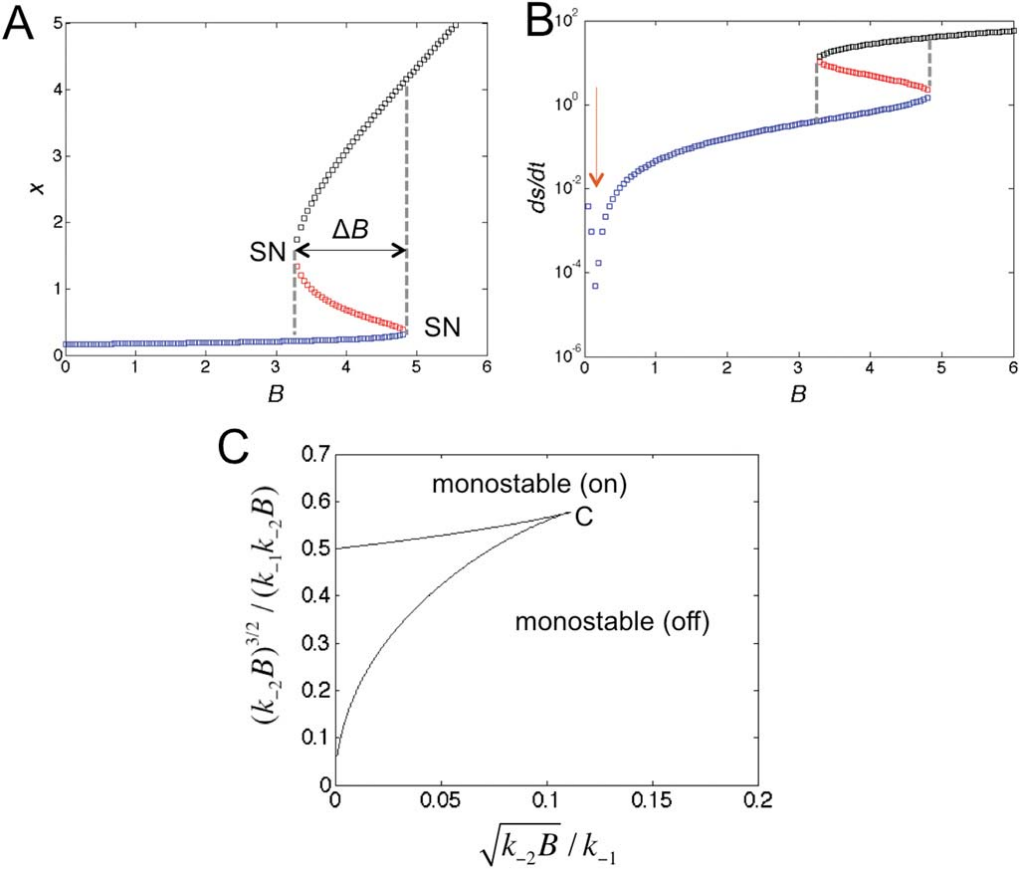
Properties of macroscopic bistable system. (A) Bifurcation diagram *x*(*B*) with the low stable steady state in blue, the unstable steady state (saddle point) in red, and high stable steady state in black for standard parameters defined in Materials and Methods. Black arrow indicates bistable regime. (B) Corresponding entropy production rate as defined in Equation 2. (C) Phase diagram showing monostable states (only low or high state) and bistable regions in *β*-*γ* plane with combination parameters *β* = *k*_−1_ *k*_+2_/(*k*_−2_ *B*)^2^ and $\gamma =k_{+1}Ak_{+2}^{2}/(k_{-2}^{3}B)$. Two phase diagrams correspond to *v* = (*k*_−2_ *B*/*k*_+2_)*V* given by 37 (exemplified by combination *V* = 10, *B* = 3.7 and standard parameters; black lines) and ∞ (red lines). The latter corresponds to the macroscopic mean-field model. SP indicates point (*β*, *γ*) = (0.22, 0.14) corresponding to standard parameters with *B* = 3.7 (see S1 Text and [31] for details).

In the limit of large volume and hence large molecule numbers, the Schlögl model is described by ODE

$$\begin{array}{c} \frac{dx}{dt}=-\underset{w_{+2}}{\underset{︸}{k_{+2}x^{3}}}+\underset{w_{-2}}{\underset{︸}{k_{-2}Bx^{2}}}-\underset{w_{-1}}{\underset{︸}{k_{-1}x}}+\underset{w_{+1}}{\underset{︸}{k_{+1}A}} \end{array}$$ (1)

with *x* the molecular concentration. Once this limit is taken, time can be sent to infinity. The resulting steady-state bifurcation diagram is shown in Fig 2A for standard parameters (see Materials and Methods), with concentration *B* chosen the control parameter. Two saddle-node bifurcations (SNs) indicate the creation/destruction of steady states, with a range of bistability described by Δ*B* in between. However, the macroscopic perspective makes no prediction about the relative stability of the two stable steady states (black and blue curves with the unstable steady state shown in red). In particular, do transitions simply become rarer with increasing volume so that the state attractors become increasingly deep but the relative weights of the states intact, or do the weights of the states change as well, leading effectively to loss of bistability? Furthermore, is there a thermodynamic selection principle for the most stable steady state? According to the second law of thermodynamics entropy is maximized in a closed system at equilibrium. Does a similar extremal principle hold for nonequilibrium steady states? The rate of entropy production describes how much heat is dissipated per time at steady state (and hence is a lower bound of how much energy is consumed to maintain the state [13, 30]):

$$\begin{array}{c} \frac{ds}{dt}=\sum_{i=1}^{2}\left(w_{+i}-w_{-i}\right)\log\left(\frac{w_{+i}}{w_{-i}}\right)\geq 0\cdot \end{array}$$ (2)

Intuitively, the entropy production is the net flux (difference between forward and backward fluxes) times the difference in chemical potential between products and educts (log term), summed over all the reactions. Equation 2 thus effectively describes how quickly the maximum entropy state is reached, if left to equilibrate. Prigogine and co-workers argued for a minimal rate of entropy production, at least near equilibrium [32], while others argued for maximal rate of entropy production [33, 34]. Fig 2B shows the macroscopic rate of entropy production. The red arrow indicates equilibrium at which the rate is zero. Notably the high state always has the higher entropy production. This can easily be understood [35] since overall in the Schlögl model *A* is converted to *B* (and vice versa, see Fig 1F). Hence, *ds*/*dt* = Δ*G*/*T* ⋅ *dA*/*dt* *x* with Δ*G* the change in free energy for the overall reaction, *T* the temperature of the bath and *dA*/*dt* a linear function of *x*. As a result, if minimal entropy production is the rule, then the low state should be selected, while maximal entropy production would dictate that the high state is more stable. As eigenvalues of the Jacobian only contain information about local stability of a fixed point and not about global stability across multiple fixed points [17, 36], further discussion needs to be postponed until the next section. Fig 2C summarizes the phase diagram (see S1 Text), showing monostable (low or high state only) and bistable regions in line with a cusp catastrophe. Limit *V* → ∞, shown by red lines, is relevant for the macroscopic description.

### Microscopic well-mixed perspective

**Fig 3:**
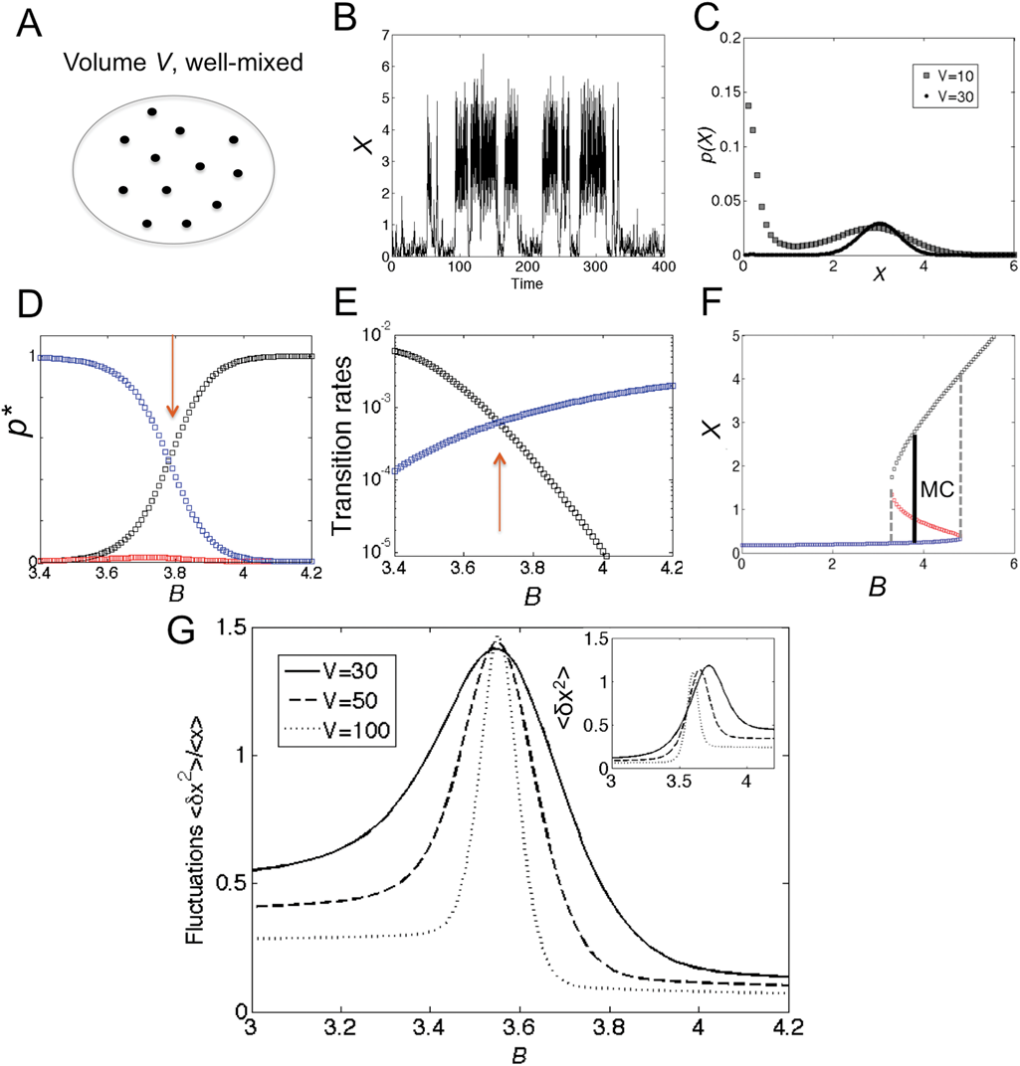
Well-mixed bistable system. (A) Schematic of well-mixed system with volume *V* (diffusion constant *D* is infinitely large). (B) Exemplar time trace for *x* = *X*/*V* from Gillespie algorithm for standard parameters with *V* = 10 and *B* = 4.0. (C) Exact probability distribution *p*(*x*) at steady state from master Equation 3 for *V* = 10 (dark symbols) and 30 (light symbols) with *B* = 4.0. (D) Values of *p*(*x*) evaluated at three steady states for different values of *B*. (E) Transition rates from a modified Fokker-Planck approximation valid for large *V* (first-mean passage time; see S1 Text for details). Red arrows indicate exchange of stability. (F) Maxwell-like construction (MC), indicating coexistence between two phases (low and high states) at *B* ∼ 3.7, defined by equal transition rates in (E). At this critical value of *B* a first-order phase transition occurs (see S1 Text for an analytical derivation based on simpler potential). (G) Relative strength of fluctuations (standard deviation over mean) as a function of *B* for *V* = 30 (solid line), 50 (dashed line), and 100 (dotted line). (Inset) Unnormalized variances.

When first taking the long-time limit for a fixed finite volume to obtain the steady-state distribution and then increasing the volume, we obtain a very different picture of bistability. Assuming a well-mixed microenvironment and thus neglecting diffusion (illustrated in Fig 3A), we can employ the one-step chemical master equation to describe the probability distribution in time

$$\begin{array}{c} \frac{d}{dt}p\left(X;t\right)=\sum_{i=\pm 1}^{\pm 2}\left[W_{i}\left(X-\nu_{i}|X\right)p\left(X-\nu_{i};t\right)-W_{-i}\left(X|X-\nu_{i}\right)p\left(X;t\right)\right] \end{array}$$ (3)

with *X* the molecule copy number and *W*_+1_(*X*∣*X* + 1) = *k*_+1_ *AV*, *W*_−1_(*X*∣*X* − 1) = *k*_−1_ *X*, *W*_+2_(*X*∣*X* − 1) = *k*_+2_ *X*(*X* − 1)(*X* − 2)/*V* ^2^, and *W*_−2_(*X*∣*X* + 1) = *k*_−2_ *BX*(*X* − 1)/*V* the volume-dependent transition rates. In Equation 3, the sum is over both the forward (*i* = +1, +2) and backward (*i* = −1,−2) reactions with *ν*_±1_ = ±1 and *ν*_±2_ = −*ν*_±1_ [30]. Using this description, we first simulate the master equation using the Gillespie algorithm [37] and confirm stochastic switching between low and high stable states (Fig 3B). However, at steady state setting *dp*/*dt* = 0, the probability distribution can analytically be derived using a recursive relation leading to *p*(*x*) = *N*(*x*) exp[−*V*Φ(*x*)] with potential [22]

$$\begin{array}{ccc} \Phi (x) & = & x\left(\ln x-1\right)+x\ln\left(\frac{k_{+2}x^{2}+k_{-1}}{k_{-2}Bx^{2}+k_{+1}A}\right)\\ & & +\;2\sqrt{\frac{k_{-1}}{k_{+2}}}\arctan\left(\sqrt{\frac{k_{+2}}{k_{-1}}}x\right)-2\sqrt{\frac{k_{+1}A}{k_{-2}B}}\arctan\left(\sqrt{\frac{k_{-2}B}{k_{+1}A}}x\right) \end{array}$$ (4)

and volume-independent prefactor

$$\begin{array}{c} N\left(x\right)=\frac{k_{+2}x^{2}+k_{-1}}{Z\sqrt{x}\left[x^{2}+k_{+1}A/\left(k_{-2}B\right)\right]}, \end{array}$$ (5)

where *Z* is a normalization constant, *x* = *X*/*V* and *p*(*x*) = *Vp*(*X*) (see S1 Text for details). By construction, the stochastic potential also has minima (a maximum) at the stable (unstable) deterministic steady states (state) with

$$\begin{array}{c} \frac{d\Phi }{dx}=-\ln\left(\frac{w_{+1}+w_{-2}}{w_{-1}+w_{+2}}\right) \end{array}$$ (6)

equal to zero at steady state (*w*_+1_ + *w*_−2_ = *w*_−1_ + *w*_+2_) and *d* ^2^Φ/*dx* ^2^ having the correct sign (see S1 Text for details and S1 Fig for a plot of Φ(*x*)). Fig 3C shows indeed that the resulting distribution of *x* has the expected bimodality.

We are now in a position to address the relative stability of the steady states, in particular of the two stable states. Fig 3D shows the probabilities evaluated at the deterministic steady states, indicating a crossing of the stable states (exchange of stability) with the probability of the unstable (metastable) state consistently below the probabilities of the stable states. A more precise picture emerges when plotting the transition rates for switching between the stable steady states in Fig 3E [22], showing coexistence of the two stable states at *B* ∼ 3.7. As derived in S1 Text, the rates depend exponentially on the volume (as expected). However, due to normalization and the volume-independence of the prefactor, the more stable of the two becomes increasingly selected for larger and larger volumes, leading effectively to monostability. Fig 3F shows that a Maxwell-type construction (MC) is required to establish the point of stability exchange, well known from the classical Van der Waals gas (see S1 Text for details) [13–15]. Since the two states have different entropy productions (Fig 2B, which can also be confirmed by calculating the microscopic entropy production defined in S1 Text), we obtain a discontinuity at this point, indicative of a first-order phase transition. Fig 3G shows indeed a sharpening of the molecular fluctuations at the critical point for increasing volume. Hence, mesoscopic cells can loose their ability for bistability (*i.e.* a bimodal distribution) with increasing volume.

The strong volume-dependent of bistability can also be seen in the phase diagram in Fig 2C (see [31]). For small volumes (black lines) the region of bistability can significantly deviate from the corresponding region in the macroscopic limit (red lines). For instance, a point in parameter space with strong bistability in the microscopic system (*B* = 3.7 for *V* = 10) is borderline bistable in the macroscopic limit (cf. Fig 3C for *B* = 4.0). However, the phase diagram does not contain information on the weights, and so shows a large bistable region even in the macroscopic limit.

### Microscopic perspective with diffusion

**Fig 4:**
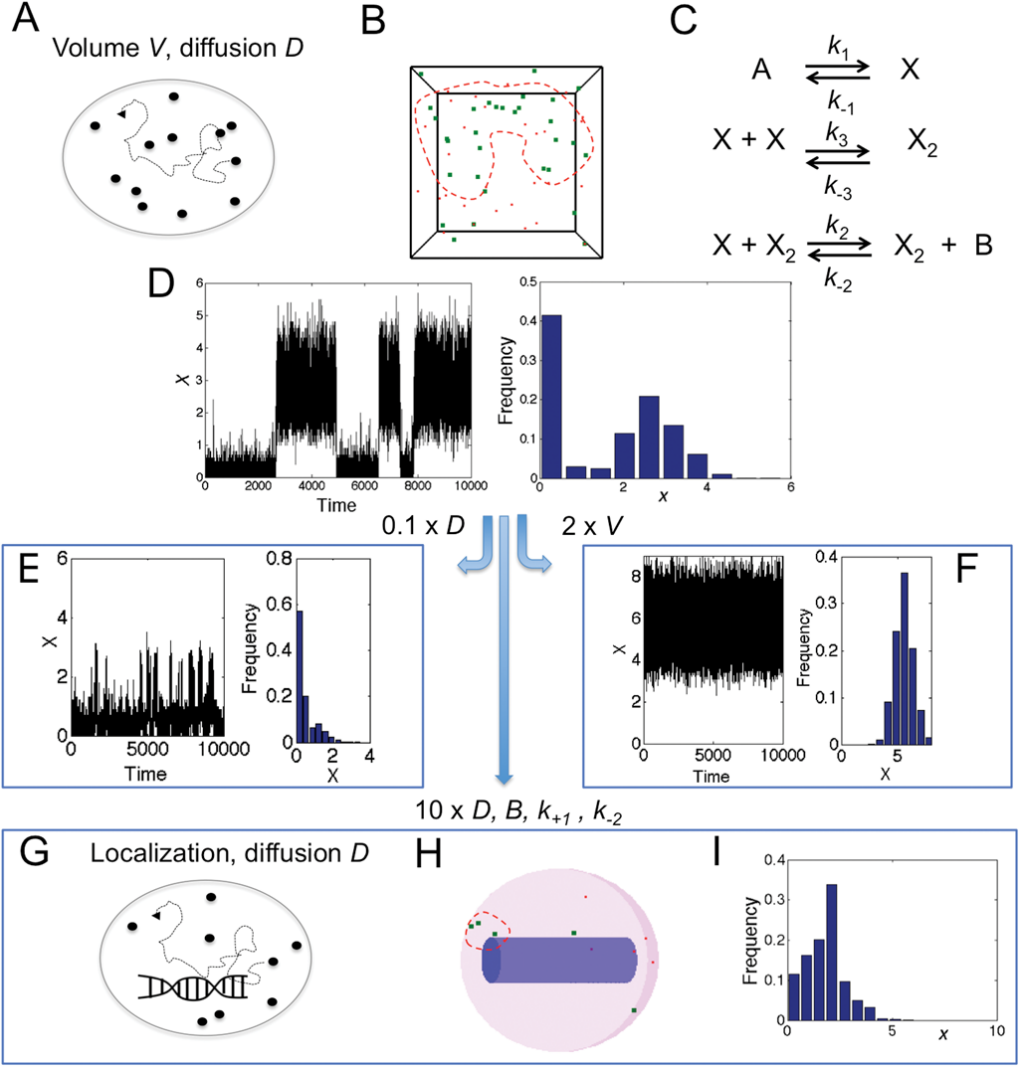
Bistable system with diffusion. (A) Schematic of diffusing molecules in volume *V*. (B) Snapshot of cubic reaction volume for generalized Schlögl model as simulated with *Smoldyn* software [38]. Shown are monomers *X* in red and dimers *X*_2_ in green. Clustering is illustrated by red dashed outline. (C) Chemical reactions of generalized Schlögl model. (D) Time trace (left) and histogram (right) of *x* = *X*/*V* from simulation for *D* = 3 (for *X*) and 1 (*X*_2_), *V* = 10, *k*_+3_ = *k*_−3_ = 1, and *B* = 3.7. (E) and (F) Effects of reduced (times 0.1) diffusion constants (E) and increased (times 2) volume (F). In (E) *B* = 3.1 to achieve comparable weights of low and high states. (G) Schematic of localized transcription in self-activating gene pathway. (H) Snapshot of spherical reaction volume with cylindrical DNA (purple) as simulated with *Smoldyn*. Shown are monomers in red and dimers in green with illustration of clustering by red dashed outline. (I) Histogram of monomer concentration *x* from simulation for *V* = 2.14 and *V*_DNA_ = 1.51, *D* = 30 (*X*) and 10 (*X*_2_), *k*_+1_ = *k*_+2_ = 50 and *B* = 50.

Diffusion introduces inhomogeneous distributions of molecules, with diffusion particularly slow in the crowded intracellular environment (Fig 4A). For this purpose we turn to the stochastic *Smoldyn* simulation package for implementing particle-based reaction-diffusion systems in a box (Fig 4B; see [38] and Materials and Methods for further details). The third-order reaction (see Fig 1F) needs to be converted into two second-order reactions since no two events can exactly occur at the same time. (We call this model the generalized Schlögl model.) This conversion requires introducing of a dimer species *X*_2_ with additional rate constants *k*_+3_ and *k*_−3_ as illustrated in Fig 4C. For *k*_+3_ = *k*_−3_ the steady-state values remain unchanged in the macroscopic limit (see S1 Text). For reasonable diffusion constants (see Materials and Methods for parameter values), we indeed observe stochastic switching, resulting in a bimodal distribution for species *X* (Fig 4D). We then compared *Smoldyn* simulations in detail with Gillespie simulations of the generalized and conventional reaction systems, including convergence for rare states with increasing simulation time, as well as effects of diffusion and dimerization reactions on bimodal distribution (see S1 Text and S2–S4 Fig). From these tests we conclude that *Smoldyn* simulations of the generalized system accurately produce bistable behavior, allowing us to study the effects of diffusion and volume on bistability.

Decreasing the diffusion constants of both molecular species by an order of magnitude, suitable for macromolecular complexes or membrane-bound proteins [39], leads to strongly fluctuating molecular concentrations (illustrated by the molecule cluster enclosed by red dashed line in Fig 4B) and reduced molecule numbers in the high state (Fig 4E). When instead increasing the reaction volume by just a factor 2, the high state is strongly induced (Fig 4F). This result resembles the destruction of bistability observed in the macroscopic limit (Fig 3D and 3F).

In Fig 4A and F the molecules are able to react anywhere in the reaction volume. However, in cells, e.g. for a self-activating gene, transcription occurs localized at the DNA molecule (Fig 4G). To investigate the effect of localization on bistability we use a spherical cellular compartment (representing e.g. a bacterial cell or a eukaryotic nucleus) in which we introduce a small cylinder to represent the DNA molecule. The production can only occur in this cylinder (Fig 4H). In contrast, degradation can occur anywhere in the cellular compartment. Fig 4I shows that bistability is destroyed with localized production, even for drastically increased production rates and diffusion constants, which would easily produce bistability under well-mixed circumstances. The broad distribution in Fig 4I may thus be caused by strong local fluctuations in molecule number (illustrated by molecule cluster enclosed by red dashed line in Fig 4H). Note that the appearance of DNA as a single copy is markedly different from the conventional or generalized Schlögl model, in which the molecule numbers scale with volume. Next, we will explore the reasons for the breakdown of bistability with inhomogeneity.

**Fig 5:**
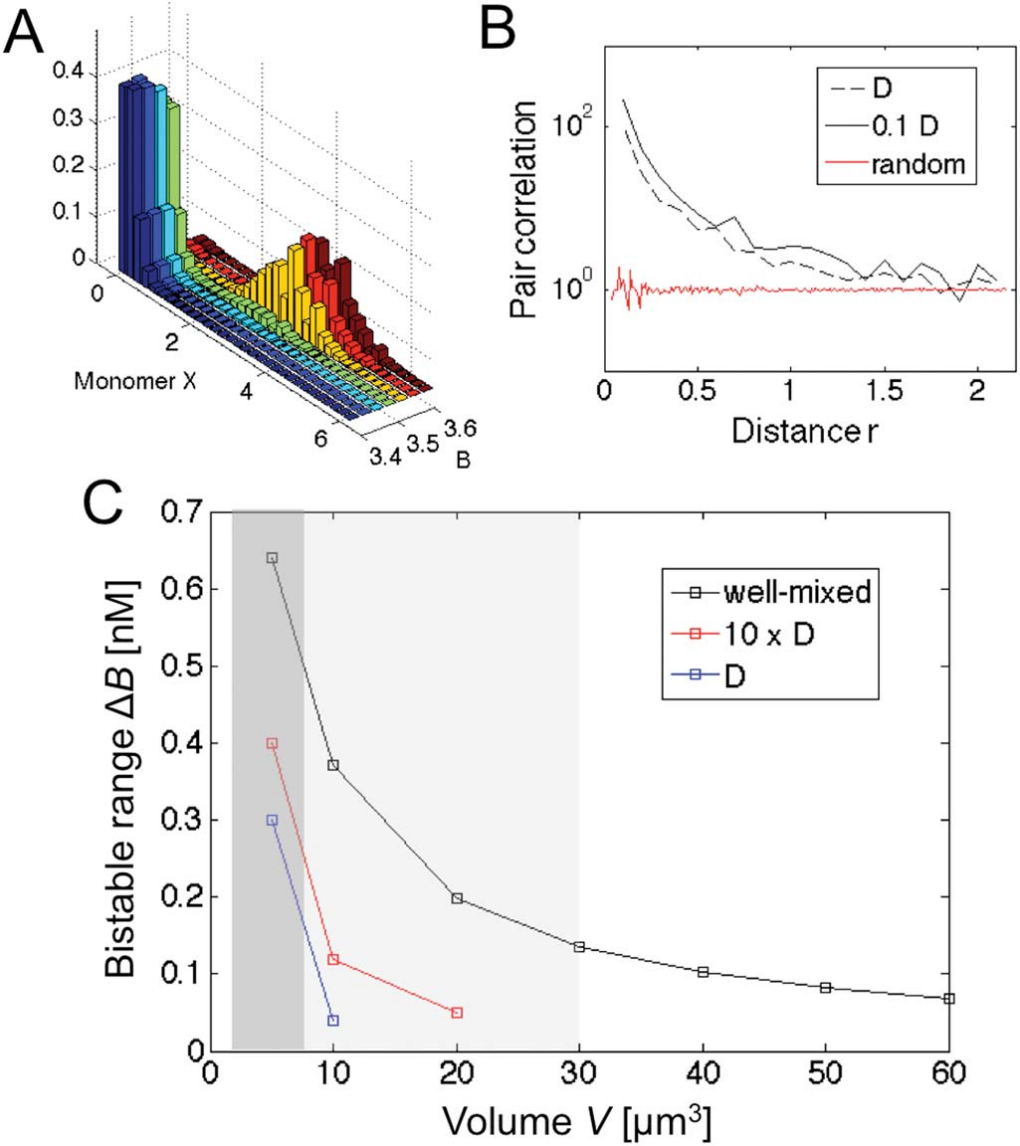
Fragility of bistability. (A) Histograms of monomer concentration *x* as a function of control parameter *B* (from 3.4 to 3.9 in steps of 0.1) with other parameters chosen as in Fig 4D. (B) Radial pair-correlation function for *D* = 3 (*X*) and 1 (*X*_2_) (dashed black line) and *D* = 0.3 (*X*) and 0.1 (*X*_2_) (solid black line) compared with random distribution (red line; see Material and Methods for details). (C) Range of *B* values with visible bimodal distribution from master equation (well-mixed, black line) and (inhomogeneous) *Smoldyn* simulations for *D* = 3 (*X*) and 1 (*X*_2_) (blue line) and *D* = 30 (*X*) and 10 (*X*_2_) (red line) in units of *μm* ^2^/*s*, the latter being typical protein diffusion constants in the cytoplasm. System was classified bistable when 10,000-long simulations (see Materials and Methods) started in low and high states showed at least one reversible switch. Note that parameters are converted to physical units here (see Materials and Methods for details). Hence, a 10,000-long-simulation corresponds to a duration of 2.78h, which is a very conservative estimate of cell-division times in bacteria and yeast. Shaded areas indicate bacterial (dark) and eukaryotic nuclear (light) volumes for comparison.

Fig 5 shows a systematic exploration of bistability from diffusive *Smoldyn* simulations, conducted similar to Fig 4D. Fig 5A shows little evidence of bistability with the system either in the low or high state. Diffusion causes strong fluctuations in molecule numbers (and hence clustering) as as demonstrated by the radial pair-correlation function *g*(*r*) in Fig 5B (not to be confused with the spike in fluctuations at the critical point of the well-mixed system in Fig 3G). For small molecule-molecule distances *r*, we obtain *g*(*r*) ≫ 1, which is the larger the slower diffusion. In contrast, random distributions of molecules do not show clustering. While the next section investigates the role of such fluctuations in the loss of bistability, our findings are summarized in Fig 5C, which shows the bistable range Δ*B* for the well-mixed case and the inhomogeneous case with finite diffusion constants. Here the system is considered bistable if simulations started from low and high states exhibit at least one reversible switch within the simulation time (see figure caption for additional details). The narrow range, especially for finite diffusion constants, suggests that bistability is a fragile property, which needs protection. Indeed, bacterial cell volume and nuclear volume in eukaryotic cells are tightly regulated (e.g. nuclear volume does not simply scale with DNA content [40]). When converting to physical units, our predicted bistability regions fall nicely into experimentally observed cell volumes (shaded areas in Fig 5C). Importantly, as volume varies during cell growth and division, such changes in volume may function as a pacemaker or trigger for phenotypic switching.

### Mechanism of bistability reduction by diffusion

Increasing the volume shifts the weights of the states leading to an effective loss of bistability (although the minima of the stochastic potential coincide with the deterministic model for sufficiently large volumes). How does slow diffusion affect bistability? There are two main potential reasons for the reduction of bistability with diffusion: (1) Diffusion may increase the barrier of switching so that bistability is harder to achieve or observe, both in simulations and experiments, or (2) diffusion may destabilize one of the stable states. In these mechanisms local fluctuations in molecule numbers may play a role as well, e.g. by introducing damaging heterogeneity or by nucleating traveling waves so that the more stable state can spread effectively and surpass the unstable state.

**Fig 6:**
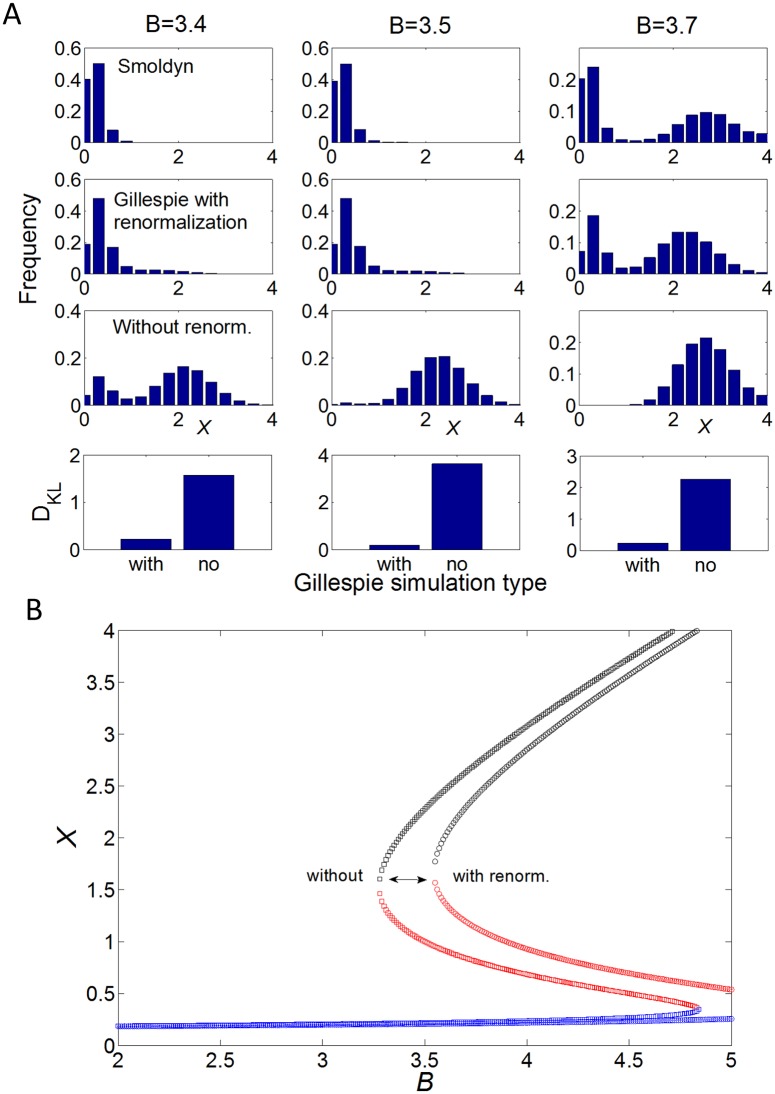
Diffusion can be included by renormalization of second-order rate constants. (A) Histograms of monomer frequency for different *B* values from simulations with *Smoldyn* software [38] (first row) and Gillespie simulations of the generalized Schlögl model with (second row) and without (third row) renormalized rate constants of second-order reactions. Standard parameters were used with volume *V* = 10. The Kullback-Leiber divergence (*D*_*KL*_) shows the closer correspondence of the renormalized reactions than the normal reactions with *Smoldyn* (forth row). For details on renormalization and calculation of *D*_*KL*_, see Materials and Methods. (B) Corresponding macroscopic bifurcation diagram of deterministic ordinary-differential equation model using renormalized rate constants *k*_±2_ to illustrate effect of diffusion. This shows that diffusion delays entry into bistable regime for increasing *B*.

To rule out (1) longer and longer simulations can be conducted to guarantee convergence. S2 Fig. shows indeed that simulations are well converged, even for weakly populated states. This shows that diffusion does not significantly change the barrier height. To investigate (2) we use the method from [41] to renormalize the second-order rate constants of the generalized Schlögl model (*k*_±2_, *k*_±3_; cf. Fig 4C) by diffusion (see Materials and Methods). This allows us to effectively include diffusion in the well-mixed model without having to conduct particle-based simulations. Fig 6A shows that histograms from Gillespie simulations of the well-mixed Schlögl model with renormalized reactions match well results from *Smoldyn* simulations (small Kullback-Leibler divergence). In contrast, Gillespie simulations without renormalized reactions do not match well. In particular, without renormalization the switch to the high state occurs at smaller *B* values. Thus, achieving bistability is easier without diffusion as it requires less thermodynamic driving. These results are summarized in Fig 6B by the bifurcation diagram of the macroscopic model of the conventional Schlögl model (Equation 1) with renormalized rate constants *k*_±2_ (note *k*_±3_ do not affect the steady-state probability distribution as they are equal, see S1 Text). Specifically, this figure shows a significant delay in achieving bistability with increasing *B* value. Completely removing first and second terms in macroscopic Equation 1 leads to the complete collapse of bistability and a truly monostable state around *x* = *k*_+1_ *A*/*k*_−1_ ≈ 0.17 in line with simulations (see S5 Fig).

**Fig 7:**
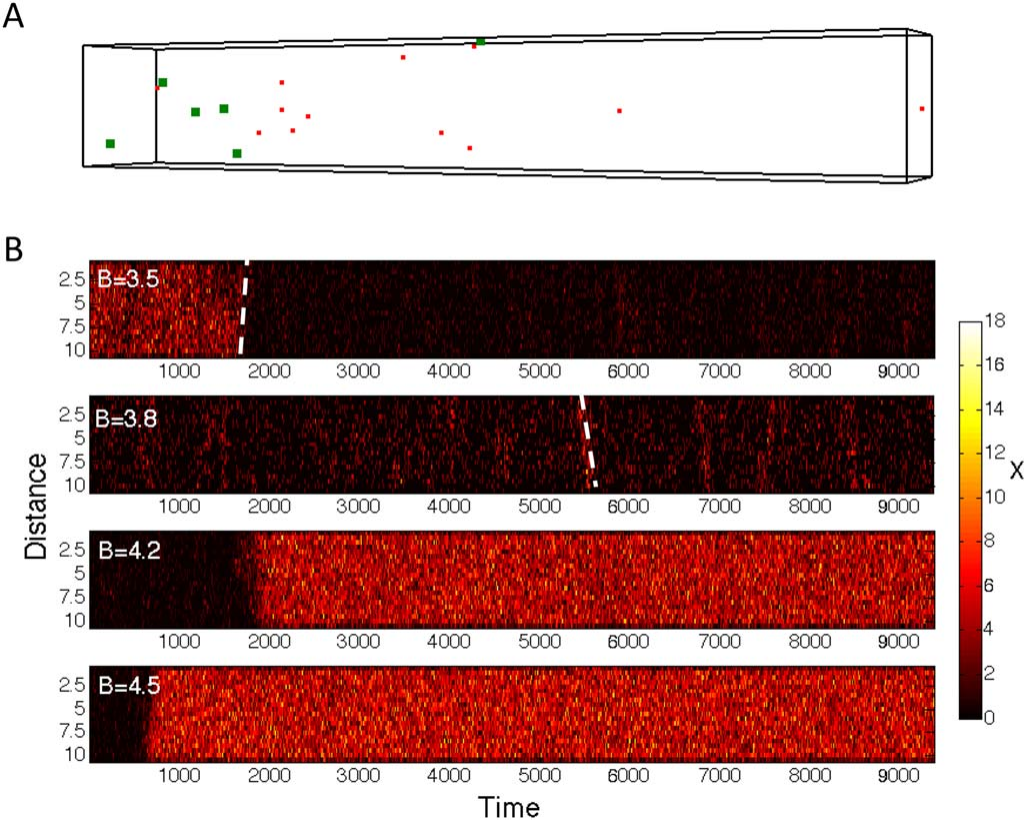
Onset of traveling waves in spatially extended system. (A) Snapshot of elongated reaction volume for generalized Schlögl model as simulated with *Smoldyn* software [38]. Shown are monomers *X* in red and dimers *X*_2_ in green. (B) Kymographs of monomer numbers along major axis of simulation box (distance) as a function of simulation time. For this purpose box was divided into 20 equal sized bins. Parameter values: Standard parameters were chosen with volume of simulation box *V* = 10 ⋅ 1.5 ⋅ 1.5, *B* values as indicate in subpanels of (B), and other parameters as in Fig 4D. Steepness of white dashed lines illustrates magnitude of wave velocity.

What role might the fluctuations observed in Figs 4B and 5B play? Following ideas from extended bistable spatial systems [16, 17], fluctuations may nucleate traveling waves, which then spread by diffusion. Although our main interest are small systems most relevant to cell biology, we extended the simulation box in one of the spatial directions (Fig 7A). Kymographs from simulations with standard parameters, run for different *B* values, show the spreading of the more stable state when initially started in the unstable state. Near co-existence at *B* ∼ 3.7, traveling waves exist which do not change the state permanently, but ripple through the box. Although wave velocities can technically be obtained from the slope in the kymographs they are highly variable and hard to determine objectively due to small molecule numbers.

Taken together, slow diffusion makes reaching bistability harder as second- (and higher-order) reactions are impaired—molecules have difficulties encountering each other to produce nonlinear behavior. Fluctuations may lead to traveling waves in more extended spatial systems, which provides a mechanism for the more stable state to overtake the less stable state.

### Experimental prediction on switching with cell-volume changes

Our models make strong predictions on the effect of cell volume. One obvious prediction is that when a system is tuned towards the bistable regime (which becomes harder and harder to achieve for increasing volume), switching between the two states becomes increasingly rare. This is the well-known transition from the stochastic to the macroscopic, deterministic limit, and was recently demonstrated using time-lapse microscopy. In budding yeast (*Saccharomyces cerevisiae*) the variability in the G1 phase (i.e. the time from division to budding) is reduced with increased ploidy (copies of chromosomes) [42]. Similarly, the switching time for turning the Pho starvation program off under reversal of phosphate limitation is reduced with ploidy [25]. As the volume also scales with ploidy, the protein concentrations stay approximately constant, thus reducing cell intrinsic noise and hence stochastic transitions between cellular states.

**Fig 8:**
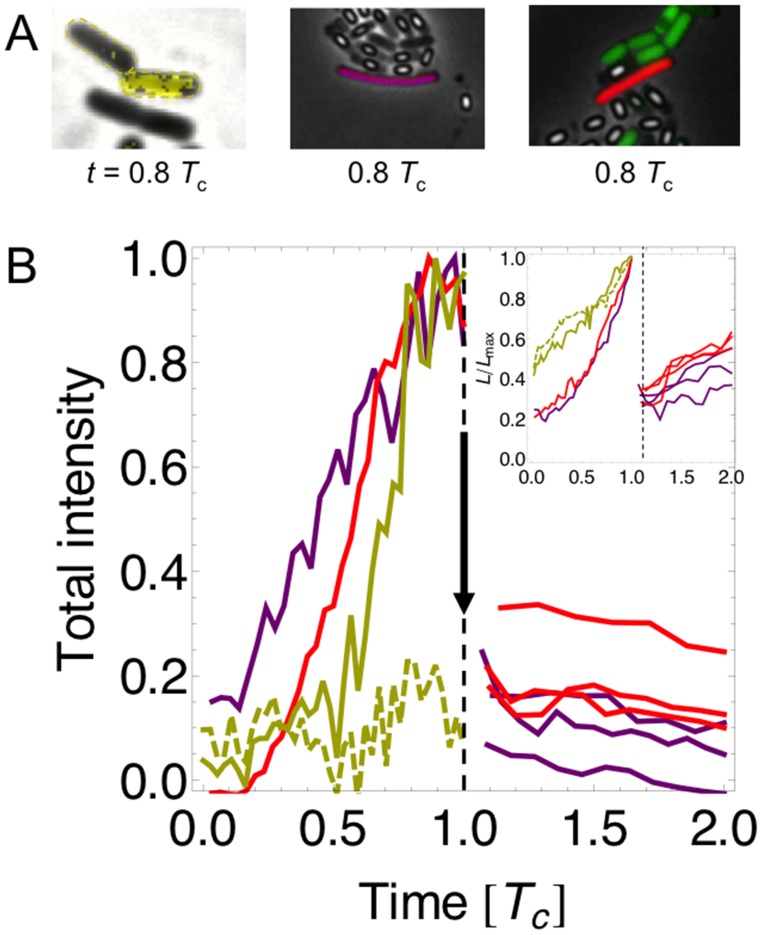
Switching may be triggered by cell-volume changes. (A) Snapshots from time-lapse fluorescence microscopy: (left) lacY-gfp of *E. coli* in yellow [43], (middle) PcomK-cfp of *B. subtilis* in purple, and (right) PcomG-cfp of *B. subtilis* in red [2] with time in units of cell-cycle time *T*_*c*_. (B) Total fluorescence intensities inside cell contours normalized to the maximal observed total intensity of a cell (see Materials and Methods for details) with color-coding same as in panel (A). Two yellow daughter cells are shown by solid and dashed lines. Note also the appearance of multiple red and purple daughter cells right after cell division in competence. (Inset) Normalized cell lengths over time in units of maximal cell length *L*_max_. S6 Fig shows same for intensity density, *i.e.* total intensity divided by cell area.

Our results, however, are more specific. They suggest increased unidirectional switching and hence monostability and decision-making in growing and dividing cells. In fact, growth towards cell division leads to a volume increase by a factor two, which may cause cells to select a steady state (see Fig 4F). Hence, for cellular parameters below the critical point, the low state will be selected, while for cellular parameters above the critical point, the high, induced state will be selected. Fig 8A and 8B show the expression of bistable reporters as a function of time, specifically LacY in *E. coli* [43] and ComK (or ComG) in *B. subtilis* [2]. The latter is indicator for competence (while competence is strictly speaking an excitatory pathway, the core module of ComK is bistable with an exit mechanism based on ComS [2]).

Fig 8B shows that switching to the high state appears during growth, while switching to the low state occurs immediately after cell division when the volume has shrunk suddenly to half its value. Note it is unlikely that the rise (drop) in fluorescence intensity is simply due to switching induced by gene duplication (halving) as the concentrations stay roughly constant due to accompanying volume changes. Furthermore, the ratio of chromosome-replication and cell-division times is known to be about 2:1 [44, 45]. Since, by visual inspection, cell division takes about 10% of the cell-cycle time (*T*_*c*_) in the time-lapse movies, chromosome replication takes about 20% of it. This duration is short compared to the rise-in-intensity phase, suggesting a different mechanism. Switching is still stochastic as shown by the two yellow daughter cells—one induces the lac operon, while the other does not. In support for our proposed scenario, spontaneous switching is extremely rare (for the lac operon estimated to be around 0.004 per cell cycle in presence of 40*μ*M inducer TMG [46]). Hence, volume changes during cell growth and division may instead be the main drivers, like a pacemaker, for switching.

## Discussion

We presented a nonequilibrium thermodynamic model of bistability, relying on molecular stochasticity and chemical energy for switching and decision-making. To cover a large class of bistable systems, including self-activating genes with cooperativity and phosphorylation-dephosphorylation cycles, we mapped minimal models for these onto the well-characterized nonequilibrium Schlögl model. Bistability and its hallmark of hysteresis are generic behaviors that are the same from one system to the next regardless of details. Indeed, this property is shared with ferro-magnets and mutually repressing genes (toggle switch) [10, 47]. Our approach is markedly different from recent deterministic approaches to postulate multistability in signaling cascades, which neglect the physical effect of cell volume and molecular diffusion [48]. Deterministic approaches often predict complex dynamics with multiple attractors. However, when the volume is sufficiently large, such behaviors can disappear. Not only does switching become increasingly rare, but also the weights shift and ultimately favor one of the states. Hence, bacterial cells and eukaryotic nuclei, and cell compartments in general, may represent protectorates of complex bi- and multistable behavior [47]. In contrast, mesoscopic cells are “boring”, unable to display complex behavior.

Slow diffusion, caused by molecular crowding and localization, is a killer of bistability and cells need to deal with this issue. This is because slow diffusion selectively penalizes second- and higher-order reactions and hence nonlinearity. Consistent with our study, ultrasensitivity in MAPK cascades is destroyed for slow diffusion due to rebinding of enzymes to their substrate [49], stressing the fundamental importance of diffusion in theoretical predictions of bistability. Phase domains and their movement are well known from the Ginzburg-Landau equation for phase transitions—this equation is in fact similar to the Schlögl model with diffusion (albeit in absence of stochastic effects). How can cells cope with the negative effects of diffusion? While adjustment of diffusion constants is difficult [50], cells could use small transcription factors to speed up diffusion. Up to about 110 kDa, the mean diffusion coefficient falls close to the Einstein-Stokes prediction for a viscous fluid [50]. This suggests that proteins up to this size do not encounter significant diffusion barriers due to macromolecular crowding or a meshwork of macromolecular structures in the cytoplasm. Indeed, the repressor LacI of the *E. coli* lac system, master regulator ComK of the *B. subtilis* competence system, and transcription factor Gal80 of the gal system in budding yeast are only 38.6, 22.4, and 48.3 kDa large, and hence are expected to have relatively large diffusion constants of at least 8*μm* ^2^/*s* (based on scaling relation in [51]). Another option for the cell is to tune the viscosity of its cytoplasm below a glass-transition point where metabolism-driven active mixing produces superdiffusive environments [52]. Note, however, that cells need to protect themselves from sudden shrinkage of the cytoplasm or drastic increases in concentration by osmotic shock.

Due to small volumes and slow diffusion in cells, bistability can only occur in a narrow range of parameter space and thus may require fine-tuning [53] or a pacemaker. Consistent with this notion, our analysis of time-lapse microscopy movies shows that volume changes during the cell cycle may trigger switching events (Fig 8). Such assistance might be necessary since spontaneous switching can be extremely rare, likely caused by rare bursts in gene expression [43, 46, 54]. For instance, the switching rate of lac system was estimated to be only 0.004 per cell cycle (in presence of 40*μ*M inducer TMG) [46], and diauxic shifts take on average 2 hours [51] (see [9, 55, 56] for additional examples of slow switching much beyond the cell cycle period). Taken together, switching may hence be more likely to occur via a thresholding mechanism [53] as implied by the Maxwell-type construction. To clarify the details of the trigger mechanism, more experimental investigation will be needed using modern microfluidic designs for continuous imaging of cells over very long times.

While some of the issues raised here have individually been discussed for the Schlögl model before [13–17], this has hardly been done in the context of biology. In particular, bistability and diffusion restrict volumes to the sizes of bacteria or eukaryotic nuclei, and volume changes during the cell cycle may be exploited to robustly change cellular states. Our particle-based simulations focus on small 3D volumes, most relevant to cellular nuclei or cytoplasms, and hence are markedly different from recent studies [16, 17]. The latter addressed role of volume and diffusion on bistability in extended 1 and 2D lattice models, respectively. Our loss of bistability for slow diffusion is largely determined by renormalization of second-order rate constants, which ultimately leads to a collapse of the system to the low state for very small diffusion constants. When extending the system in one of the spatial dimensions, we observe the onset of traveling waves, which may ultimately determine switching rates for even larger systems. Specifically, for extended systems the wave velocity is determined by the deterministic (and not the stochastic) potential [16, 17]. Such quasi-1D extended states may biological be relevant in filamentous bacterial cells. Traveling waves are indeed observed in *Smoldyn* simulations of the Min-system, a small biochemical pathway which allows cells to determine their middle for accurate cell division [57].

Bistability is fascinating due to its connection with nonequilibrium physics, first-order phase transitions, and decision-making in cells. Our results show that *volume shifts the weights* of the states relative to each other but not the steady-state values directly. Below a critical value the low state is selected, while above a critical value the high state is selected. In contrast, *diffusion shifts the steady-state values*. For sufficiently slow diffusion, only the low state survives. While widely studied there are a number of open questions. One pressing question is whether epigenetic information is inherited in the spirit of Hopfield’s content-addressable memory [58]. Since the seminal work by Novick and Weiner in 1957, such inheritance seems indeed to apply to the Lac system [59], while Fig 8 shows that the daughter cells do not generally inherit the competence state (this might be due to the protease MecA [2]). Furthermore, cell volume changes and their effect on bistability also have many biomedical implications. Examples include viruses such as bacteriophage lambda [55] and HIV [60], as well as pancreatic *β* cells, responsible for glucose sensing and insulin production. These cells undergo large size changes, e.g. during pregnancy [61]. Thermodynamics may shed new light on their regulatory mechanisms.

## Materials and Methods

### Schlögl model

In 1972 Schlögl proposed two chemical reaction models for nonequilibrium phase transitions [14]. One example shows a phase transition of first order, while another shows a phase transition of second order. When diffusion is included in the the first-order transition, coexistence of two phases in different spatial domains may occur. For spherical domains the coexistence indicates the onset of the transition similarly to the vapor pressure in droplets or bubbles. The volume dependence has been discussed early, e.g. in [31]. The related Keizer’s paradox has mostly been discussed in context of Schlögl’s first model, but also for the logistic growth equation [21], showing its relevance to a wide sectrum of systems. In these systems, large rare fluctuations have severe consequences. Keizer’s paradox says that deterministic and stochastic approaches can lead to fundamentally different results. In particular, the deterministic model considers the infinite volume limit (*V* → ∞) before considering the steady-state limit (*t* → ∞). Hence, no transitions between steady states are allowed. The state of the system only depends on the initial condition, which seems unphysical. In contrast, the stochastic model, by taking the steady-state limit first, can always settle in its lowest state, and thus is ultimately favored over the deterministic approach. Gaspard [30] and later Qian [13] took the perspective of open chemical system, and analyzed the second Schlögl model in terms of fluxes and entropy production. The Schlögl model can be considered the simplest bistable system but has not yet been verified or implemented experimentally by suitable chemical reactions.

### Implementation and parameters

The chemical reactions of the Schlögl model can be found in Fig 1F. Macroscopically (for an infinite volume) this model can be described by ODE Equation 1. For finite volume but infinitely fast diffusion (well mixed case) the master equation (Equation 3) can be used or Gillespie simulations [37]. For the solution of the master equation and a derivation of the transition rates see S1 Text. The macroscopic (microscopic) entropy production formula is provided in Equation 2 (in S1 Text). The chemical reactions for the generalized Schlögl model are given in Fig 4C with further details provided in S1 Text.

Stochastic spatio-temporal simulations with diffusion were conducted with *Smoldyn* software version 2.28 as described in [38]. Briefly, it is a particle-based, fixed time-step, space-continuous stochastic algorithm for reaction-diffusion systems in various geometries based on Smoluchowski reaction dynamics [62]. Both simulations and Smoluchowski theory only apply to reactions up to second order. Given reaction rate constants, diffusion constants, and time step, *Smoldyn* determines reaction radii, *i.e.* binding and unbinding radii. The binding radii correspond to the encounter complex, formed by diffusion. Once formed, the reaction occurs. Intrinsic rate constants are strictly constant, i.e. independent of diffusion, for low particle densities and activation-limited reactions (see manual for details). This is checked and confirmed by *Smoldyn* at the beginning of each simulation. Under these conditions, the effects of rate constants and diffusion constants on bistability can independently be explored.

The renormalization of the second-order rate constants to effectively include diffusion into the well-mixed generalized Schlögl model is done following [41]. Using *k*_*D*_ = 4*πσD* to describe the encounter of two molecules by diffusion, the following rate constants are obtained

$$\begin{array}{c} k_{\pm i}^{\prime }=\frac{k_{\pm i}k_{D}}{k_{+i}+k_{D}} \end{array}$$ (7)

with *i* = 2, 3 (cf. Fig 4C), *k*_±*i*_ comparable to *Smoldyn*’s intrinsic rate constants, product of cross section *σ*, and average diffusion constant *D* set to 0.5.

Unitless parameters were generally used as given in [30], termed standard parameters. These rate constants are *k*_+1_ *A* = 0.5, *k*_−1_ = 3, and *k*_+2_ = *k*_−2_ = 1. Only concentration *B*, diffusion constants, and volume *V* were varied as indicated in figure captions. For *Smoldyn* simulations we additionally used *k*_+3_ = *k*_−3_ = 1 (Fig 4D and 4F) and *V* = 2.14, *V*_DNA_ = 1.51,*D* = 30 (*X*) and 10 (*X*_2_), *k*_1_ = *k*_2_ = 50 and *B* = 50 (Fig 4I). Simulation time was generally 10,000 unless specified differently. To convert to units in Fig 5C, we set length and time scales to respective *μm* and *s*. We further express concentration in [nM], with 1nM corresponding to 1 (1000) molecules in a typical bacterial (eukaryotic HeLa) cell, and volume as *V* = *η*10*μm* ^3^ with *η* a scaling number of order 1. Rate constants then become *k*_+1_ *A* = 5/(6*η*) [nM/s], *k*_−1_ = 1 [*s* ^−1^], *k*_+2_ = *B* [*s* ^−1^], *k*_−2_ = *k*_+3_ = 6*η*/10 [(nM s)^−1^], and *k*_−3_ = 1 [*s* ^−1^]. For gene expression, *k*_+1_ *A* corresponds to typical basal expression rates found in bacteria [63].

### Simulation analysis

The radial pair-correlation functions in Fig 5B were calculated from 10 simulation snap shots of monomer *X* positions **r**_*i*_ in a 3D cube of volume *V*, using

$$\begin{array}{c} g\left(r\right)=\frac{V}{N(N-1)}\frac{1}{4\pi r^{2}a}\sum_{i=1}^{N}\sum_{j\neq i=1}^{N}I_{ij}\left(r-a<|\right|r_{j}-r_{i}\left||\leq r\right) \end{array}$$ (8)

for *r* ≥ *a* with *a* the mesh spacing, assuming periodic boundary conditions. *I* is either one if its argument is true or zero if false. *N* is the current monomer number. For plot *g*(*r*) was then averaged over snapshots for different *N*. Note since *N* varies, *g*(*r*) can systematically deviate from 1. As a control, random positions were produced for which *g*(*r*) ∼ 1 as expected.

The comparison of distributions in Fig 6A is done with the Kullback-Leibler divergence, defined by

$$\begin{array}{c} D_{KL}=\sum_{i}p_{R}\left(x_{i}\right)\ln\left[\frac{p_{R}\left(x_{i}\right)}{p_{T}\left(x_{i}\right)}\right], \end{array}$$ (9)

where *p*_*R*_(*x*_*i*_) and *p*_*T*_(*x*_*i*_) are the reference and test distributions, respectively. The sum in Equation 9 is over bins in *x* space.

### Image analysis

Fluorescence intensities of bacterial cells were extracted from time-lapse movies of fluorescence microscopy (*E. coli* from [43] and *B. subtilis* from [2]). The active-contour method from [64] as an *ImageJ* plugin was used to detect the cell boundaries as ridges, and pixel intensities inside of the contours were collected using a simple custom written *Mathematica* code. The background intensities including the phase contrast intensities were subtracted and only the intensities from the fluorescence channels were plotted in Fig 8B. Intensity density plotted in S6 Fig is calculated as total intensity of a cell divided by the cell current focal area.

## Supporting Information

S1 Text Supporting text with mathematical derivations and additional explanations. (PDF)

S1 Fig Comparison of deterministic and stochastic potentials. For concentration *x* the deterministic potentials Ψ(*x*) in green is calculated with Eq. 44 in S1 Text, while the stochastic potential Φ(*x*) in blue is calculated with main-text Equation 4 (Eq. 19 of S1 Text). (A) Arrow indicates that for *B* = 3.0 both potentials predict the low state as most stable. (B) Arrows indicate that around *B* = 3.4 the deterministic potential predicts coexistence of the low and high states. (C) Arrows indicate that around *B* = 3.7 the stochastic potential predicts coexistence. (D) At *B* = 4.0 both potentials predict the high state as most stable.
(TIF)

S2 Fig Bimodal distribution with rare high state (low weight) for *B* = 3.5 from *Smoldyn* simulations converge for increasing simulation time. Remaining parameters were chosen as in Fig 4D with *x* the monomer concentration. (A-C) Simulation time is increased from *t* = 1,000, 5,000, to 10,000 as indicated. Arrow in (C) points to high state. (D) Kullback-Leibler divergence between each of the three simulations and *Smoldyn* simulation for *t* = 50,000 as reference distribution (see S1 Text for details).
(TIF)

S3 Fig Comparison of *Smoldyn* simulations and Gillespie simulations of generalized Schlögl model for increasing diffusion constant *D* for *B* = 3.5 and *t* = 10,000. Remaining parameters were chosen as in Fig 4D with *x* the monomer concentration. (A) Gillespie simulation. (B) *Smoldyn* simulation for *D* = 3 (*X*) and 1 (*X*_2_). (C) *Smoldyn* simulation for faster diffusion with *D* = 15 (*X*) and 5 (*X*_2_). (D) Kullback-Leibler divergence between each of the two *Smoldyn* simulations and Gillespie simulation as reference distribution (see S1 Text for details).
(TIF)

S4 Fig Distributions from *Smoldyn* simulations become increasingly similar to results from Gillespie simulations of conventional Schlögl model for increasing dimerization rate constants *k*_+3_ = *k*_−3_ as indicated, as well as fast diffusion (parameters as in S3C Fig) with enlarged *B* = 3.7. (A) Gillespie simulation for *B* = 3.2. (B) *Smoldyn* simulation for *k*_+3_ = *k*_−3_ = 1. (C) *Smoldyn* simulation for *k*_+3_ = *k*_−3_ = 3. (D) Kullback-Leibler divergence between each of the two *Smoldyn* simulations and Gillespie simulation as reference distribution (see S1 Text for details).
(TIF)

S5 Fig Drastic reduction of diffusion collapses bistabiliy to predicted low monostable state for different *B* values as indicated (see main text section “Microscopic perspective with diffusion”). Scaling factor multiplies set of diffusion constants, *D* = 3 (*X*) and 1 (*X*_2_). Remaining parameters as in S3B Fig.
(TIF)

S6 Fig Switching may be triggered by cell-volume changes. Image analysis similar to Fig 8B but with total intensity normalized by cell area to provide the intensity density.
(TIF)
