# Supplementary material for: Bistability: Requirements on Cell-Volume, Protein Diffusion, and Thermodynamics
Source: PLoS One. 2015 Apr 15;10(4):e0121681. doi: 10.1371/journal.pone.0121681 (PMC4398428; doi:10.1371/journal.pone.0121681)
Supplement: S1 Text — (PDF) [file pone.0121681.s001.pdf]

Supporting Information to

Bistability: requirements on cell-volume, protein diffusion, and thermodynamics

Robert G. Endres

Department of Life Sciences & Centre for Integrative Systems Biology and Bioinformatics, Imperial College, London, United Kingdom

In this Supporting Information we provide or derive equations used in the main text and give additional explanations. The chemical reactions considered here are given by the Schlögl model model with single species X and reactions [1]

$$A \underset{k_{-1}}{\overset{k_{+1}}{\rightleftharpoons}} X \tag{1}$$

$$3X \underset{k_{-2}}{\overset{k_{+2}}{\rightleftharpoons}} 2X + B, \tag{2}$$

where A and B are clamped concentrations, driving the system out of equilibrium, and k_{+1}, k_{-1}, k_{+2}, and k_{-2} rate constants. The latter are provided in the Materials and Methods section of the main text.

I. STEADY-STATE SOLUTION OF MASTER EQUATION

The one-step chemical master equation for chemical species X can generally be written as

$$\frac{d}{dt}P(X;t) = \sum_{i=\pm1}^{\pm2}[W_i(X-\nu_i|X)P(X-\nu_i;t) - W_{-i}(X|X-\nu_i)P(X;t)] \tag{3}$$

with the transition rates given by

$$W_{+1}(X|X+1) = k_{+1}AV \tag{4}$$

$$W_{-1}(X|X-1) = k_{-1}X \tag{5}$$

$$W_{+2}(X|X-1) = k_{+2}X\frac{X-1}{V}\frac{X-2}{V} \tag{6}$$

$$W_{-2}(X|X+1) = k_{-2}BX\frac{X-1}{V} \tag{7}$$

for reaction volume V. Note that $\nu_{\pm1} = \pm1$ and $\nu_{\pm2} = \mp1$ [2]. At steady state, the master equation can be solved exactly by iteration [3]. Introducing short notation

$$W^+(X) = W_1(X|X+1) + W_{-2}(X|X+1) \tag{8}$$

$$W^-(X) = W_{-1}(X|X-1) + W_{+2}(X|X-1) \tag{9}$$

for the combined transition rates starting from X molecules, $dP/dt = 0$ produces

$$W^+(X-1)P(X-1) + W^-(X+1)P(X+1) - [W^+(X) + W^-(X)]P(X) = 0 \tag{10}$$

and, since valid for all X, the detailed balancing equation

$$W^+(X)P(X) = W^-(X+1)P(X+1), \tag{11}$$

which can be solved by iteration for the steady-state probability distribution

$$P(X) = P(0)\prod_{i=0}^{X-1}\frac{W^+(i)}{W^-(i+1)} = P(0)\exp\sum_{i=0}^{X-1}\ln\frac{W^+(i)}{W^-(i+1)}. \tag{12}$$

Using concentrations instead of copy numbers, i.e. $x = X/V$ and scaling $p(x) = VP(X)$, this leads to

$$p(x) = N(x)\exp[-V\Phi(x)] \tag{13}$$

with the stochastic potential defined in the large-volume limit as

$$\Phi(x) = -\int_0^x dy\ln\left(\frac{\gamma^+(y)}{\gamma^-(y)}\right) \tag{14}$$

with scaling $W^\pm(X) = V\gamma^\pm(x)$ and the definitions $\gamma^+ = w_{+1} + w_{-2}$ and $\gamma^- = w_{-1} + w_{+2}$. The rates are now given by

$$w_{+1} = k_{+1}A \tag{15}$$

$$w_{-1} = k_{-1}x \tag{16}$$

$$w_{+2} = k_{+2}x^3 \tag{17}$$

$$w_{-2} = k_{-2}Bx^2. \tag{18}$$

The remaining integral in Eq. 14 can be done analytically, producing [3, 7]

$$\begin{aligned}\Phi(x) &= x(\ln x - 1) + x\ln\left(\frac{k_{+2}x^2 + k_{-1}}{k_{-2}Bx^2 + k_{+1}A}\right)\\ &+2\sqrt{\frac{k_{-1}}{k_{+2}}}\arctan\left(\sqrt{\frac{k_{+2}}{k_{-1}}}x\right) - 2\sqrt{\frac{k_{+1}A}{k_{-2}B}}\arctan\left(\sqrt{\frac{k_{-2}B}{k_{+1}A}}x\right).\end{aligned} \tag{19}$$

The volume-independent prefactor in Eq. 13 is given by

$$N(x) = \frac{k_{+2}x^2 + k_{-1}}{Z\sqrt{x}[x^2 + k_{+1}A/(k_{-2}B)]} \tag{20}$$

with Z a normalization constant. Stochastic potential Φ has indeed minima at the stable steady states of the deterministic solution and a local maximum at the unstable deterministic steady state since

$$\frac{d\Phi}{dx} = \ln\left(\frac{\gamma^-}{\gamma^+}\right) \tag{21}$$

is zero for $\gamma^+ = \gamma^-$ and

$$\frac{d^2\Phi}{dx^2} = -\frac{\frac{d\gamma^+}{dx} - \frac{d\gamma^-}{dx}}{\gamma^+} = \frac{\Delta\gamma'}{\gamma^+} \tag{22}$$

is > 0 (< 0) for stable (unstable) steady states with $\Delta\gamma' = d\gamma^-/dx - d\gamma^+/dx$. The sign of the second derivative of Φ can easily be understood when considering standard linear stability analysis. Linearizing the ordinary differential equation of the Schlögl model (Eq. 1 in main text) with $x = x_0 + \delta x$ around steady state x_0 produces

$$\frac{d(\delta x)}{dt} = -\Delta\gamma'(x_0)\delta x \tag{23}$$

with perturbations decaying exponentially for $\Delta\gamma' > 0$.

Next, we consider the transition rates between the two minima. Using a modified Fokker-Planck equation, known to correctly describe transport laws at large volume, the mean first-passage time is estimated from the splitting of the degenerate smallest eigenvalue [3, 4]. The transition rate for switching from the low (x_1) to the high (x_2) state via the unstable state (x_0) can then be calculated via

$$r^+_{1\to 2} = \frac{\gamma^+(x_1)\sqrt{-\Phi''(x_0)\Phi''(x_1)}}{2\pi V} e^{-V[\Phi(x_0)-\Phi(x_1)]} \tag{24}$$

$$= \sqrt{\frac{\gamma^+(x_1)}{\gamma^+(x_0)}} \frac{\sqrt{-\Delta\gamma'(x_0)\Delta\gamma'(x_1)}}{2\pi V} e^{-V[\Phi(x_0)-\Phi(x_1)]} \tag{25}$$

with short notation $\Phi'' = d^2\Phi/dx^2$. Similarly, the transition rate for switching from the high (x_2) to the low (x_1) state is given by

$$r^-_{2\to 1} = \frac{\gamma^+(x_2)\sqrt{-\Phi''(x_0)\Phi''(x_2)}}{2\pi V} e^{-V[\Phi(x_0)-\Phi(x_2)]} \tag{26}$$

$$= \sqrt{\frac{\gamma^+(x_2)}{\gamma^+(x_0)}} \frac{\sqrt{-\Delta\gamma'(x_0)\Delta\gamma'(x_2)}}{2\pi V} e^{-V[\Phi(x_0)-\Phi(x_2)]}. \tag{27}$$

Their ratio is given by the simpler expression

$$\frac{r^+}{r^-} = \sqrt{\frac{\Delta\gamma'(x_2)\gamma^+(x_1)}{\gamma^+(x_2)\Delta\gamma'(x_1)}} e^{-V[\Phi(x_1)-\Phi(x_2)]}. \tag{28}$$

Rates scale with system size and become exponentially small with increasing V. However, unlike the conventional Fokker-Planck equation as derived from the truncated Kramers-Moyal expansion of the master equation, our rates depend on correct stochastic potential $\Phi(x)$. Other suitable approaches are the large deviation (or eikonal or Wentzel-Kramers-Brillouin (WKB)) approximation [5].

II. MICROSCOPIC ENTROPY PRODUCTION

In the main text we use the entropy production to quantify the heat dissipation and for characterizing the degree of nonequilibrium. Following [2], the microscopic entropy production for the master equation is given by

$$\frac{dS}{dt} = \frac{1}{2}\sum_{X,i} J_i(X;t)A_i(X;t) \geq 0 \tag{29}$$

with reaction rates

$$J_i(X;t) = W_i(X-\nu_i|X)P(X-\nu_i;t) - W_{-i}(X|X-\nu_i)P(X;t) \tag{30}$$

and affinities

$$A_i(X;t) = \ln\frac{W_i(X-\nu_i|X)P(X}{W_{-i}(X|X-\nu_i)P(X;t)}. \tag{31}$$

In the macroscopic limit, this reduces to Eq. 2 of the main text, given by

$$\frac{1}{V}\frac{dS}{dt} = \frac{ds}{dt} = (w_{+1}-w_{-1})\ln\left(\frac{w_{+1}}{w_{-1}}\right) + (w_{+2}-w_{-2})\ln\left(\frac{w_{+2}}{w_{-2}}\right) \geq 0. \tag{32}$$

III. PHASE DIAGRAM

Following Ebeling and Schimansky-Geier [6], we derive a general volume-dependent phase diagram for the well-mixed conventional Schlögl model, valid even for small volumes. Extrema for discrete molecule numbers based on the master equation obey

$$P(X) = P(X-1). \tag{33}$$

Using detailed balancing from Eq. 11 this simplifies to condition

$$\tilde{W}^+(X-1) = \tilde{W}^-(X), \tag{34}$$

where tilde represents rescaled transition rates with time in units of k_{+2}^{-1}, given by

$$\tilde{W}^+(X) = \tilde{A}V\left[\frac{X(X-1)}{V^2} + \tilde{C}\right] \tag{35}$$

$$\tilde{W}^-(X) = X\left[\frac{(X-1)(X-2)}{V^2} + \tilde{B}\right] \tag{36}$$

and $\tilde{A} = k_{-2}B/k_{+2}$, $\tilde{B} = k_{-1}/k_{+2}$, and $\tilde{C} = (k_{+1}A/k_{-2})B$. As a result, Eq. 34 leads to third-order polynomial

$$-X^3 + (3+v)X^2 - (2+3v+\beta v^2)X + (2v+\gamma v^3) = 0 \tag{37}$$

with abbreviations $v = \tilde{A}V = (k_{-2}B/k_{+2})V$, $\beta = \tilde{B}/\tilde{A}^2 = k_{-1}k_{+2}/(k_{-2}B)^2$, and $\gamma = \tilde{C}/\tilde{A}^2 = k_{+1}Ak_{+2}^2/(k_{-2}^3B)$.

The system looses bistability if the discriminant disappears, i.e. when

$$D = \left(\frac{\beta}{3} - \frac{1}{9} + \frac{1}{3v} - \frac{1}{3v^2}\right)^3 + \left[-\frac{1}{27} + \frac{\beta}{6} - \frac{\gamma}{2} + \frac{1}{2v}\left(\beta + \frac{1}{3}\right) - \frac{1}{6v^2}\right]^2 = 0. \tag{38}$$

The resulting solution for γ is

$$\gamma(\beta, v) = \frac{1}{3}\left(\beta - \frac{2}{9}\right) + \frac{1}{v}\left(\beta + \frac{1}{3}\right) - \frac{1}{3v^2} \pm \frac{2}{3\sqrt{3}}\left(\frac{1}{3} - \beta - \frac{1}{v} + \frac{1}{v^2}\right)^{3/2}. \tag{39}$$

The edge of the cusp, i.e. the triple point, is given by

$$\beta_C = \frac{1}{3} - \frac{1}{v} + \frac{1}{v^2} \tag{40}$$

$$\gamma_C = \frac{1}{27} + \frac{1}{3v} - \frac{1}{v^2} + \frac{1}{v^3}. \tag{41}$$

Importantly, Eq. 39 has a strong volume dependence. For instance, in the macroscopic limit $v \to \infty$ we obtain

$$\gamma_\infty = \frac{1}{3}\left(\beta - \frac{2}{9}\right) \pm \frac{2}{3\sqrt{3}}\left(\frac{1}{3} - \beta\right)^{3/2}. \tag{42}$$

IV. MAXWELL-LIKE CONSTRUCTION

The Schlögl model is formally equivalent to the van der Waals gas and hence has a first-order phase transition, requiring a Maxwell-like construction [1]. For a particular value of control parameter B_{MC} in the bifurcation diagram, there is a coexistence of the "two phases". When the stabilities of the two stable states cross, the system exhibits a discontinuous jump in the "free energy" (to be defined below).

The coexistence is correctly defined by equal transition rates between the low and high states, which according to Fig. 3F of main text is at $B \sim 3.7$. Note this is slightly different from $B \sim 3.8$ at which the two states have the same value for the stochastic potential $\Phi(x)$, given by Eq. 19 and shown in Fig. 3E. To make analytical progress, we follow [1] and use below a simpler *ad hoc* potential $\Psi(x)$.

By writing the ordinary differential equation for the Schlögl model in the form

$$\dot{x} = -k_{+2}x^3 + k_{-2}Bx^2 - k_{-1}x + k_{+1}A = -\frac{\partial \Psi}{\partial x}, \tag{43}$$

with short notation $\dot{x} = dx/dt$, we define the deterministic potential

$$\Psi(x) = \frac{k_{+2}}{4}\left(x^4 - \underbrace{\frac{4k_{-2}B}{3k_{+2}}}_{=\alpha} x^3 + \underbrace{\frac{2k_{-1}}{k_{+2}}}_{=\beta} x^2 - \underbrace{\frac{4k_{+1}A}{k_{+2}}}_{=\gamma} x + const \right), \tag{44}$$

which has minima at the stable steady state and a local maximum at the unstable steady state. Imposing coexistence we write

$$\Psi(x) = \frac{k_{+2}}{4}(x - x_1)^2(x - x_2)^2, \tag{45}$$

where x_1 and x_2 are the two stable steady-state values. After multiplying out Eq. 45 and comparing coefficients with Eq. 44, we obtain the following conditions

$$u = 2(x_1 + x_2) \tag{46}$$

$$v = u^2/4 - 2x_1x_2 \tag{47}$$

$$w = ux_1x_2. \tag{48}$$

For given model parameters and hence u, v and w, the two stable steady states are given by

$$x_{1,2} = \frac{u}{2}\left(1 \pm \sqrt{3 - \frac{vw}{u^2}}\right). \tag{49}$$

For given x_1 and x_2, we can also derive an equation relating all the parameters to each other for coexistence

$$\frac{u}{2}\left(v - \frac{u^2}{4}\right) = w. \tag{50}$$

For instance, for the standard parameters used here, $k_{+1}A = 0.5, k_{-1} = 3$, and $k_{+2} = k_{-2} = 1$, we obtain $B_{MC} \approx 3.39$. In Fig. S1 we compare the deterministic potential with the exact stochastic potential from Eq. 19 for different B values, illustrating the difference in critical B value.

At B_{MC} there is a discontinuity characteristic of a first-order phase transition since the entropy-production rate, i.e. the dissipated free energy, is different for the two stable states [7]. For $B < B_{MC}$ the low-x minimum entropy production state is selected, while for $B > B_{MC}$ the high-x maximum entropy production state is selected. Hence, for sufficiently large V, bistability is replaced by a phase transition. Note also that this shows that neither a minimum or maximum entropy production principle describes the system for all parameters [8, 9] in contradiction to suggestions in [10]. However, the value B_{MC} is close to the threshold

value for the onset of "bistability", which is around $B \approx 3.3$, indicating that for most parameter values of B the entropy production rate is at a maximum. For the Schlögl model, the upper high-x state has always the larger entropy production rate [9].

V. GENERALIZED SCHLÖGL MODEL

In order to conduct spatio-temporal stochastic simulations of the third-order Schlögl model using program *Smoldyn* [11], we introduce an intermediate dimer species with copy number X_2 (and concentration $x_2 = X_2/V$). In terms of ordinary differential equations, the model is given by

$$\dot{x} = -k_{+2}x_2x + (k_{-2}B + k_{-3})x_2 - k_{+3}x^2 - k_{-1}x + k_{+1}A \quad (51)$$

$$\dot{x}_2 = k_{+3}x^2 - k_{-3}x_2, \quad (52)$$

where the total molecule concentration is $x_T = x + 2x_2$. Choosing new rate constants $k_{+3} = k_{-3} = k$ leads to the situation that k only determines the dynamics, that is how quickly monomer and dimer convert into each other and hence how quickly the steady state is reached, without affecting the steady state value of the monomer concentration x. At steady state, the resulting equation for the monomer concentration is equivalent to the conventional Schlögl model, i.e.

$$-k_{+2}x^3 + k_{-2}Bx^2 - k_{-1}x + k_{+1}A = 0, \quad (53)$$

allowing direct comparison between the conventional and the generalized model.

VI. COMPARISON OF *SMOLDYN* SIMULATIONS WITH CONVENTIONAL AND GENERALIZED SCHLÖGL MODELS

For the generalized Schlögl model with a rare high state for $B = 3.5$, Fig. S2 shows that the high state appears for simulations of duration of $10,000$ and more. For these simulation times the distribution of monomer concentration x converges to the correct distribution from Gillespie simulations of the generalized system as judged by the small Kullback-Leibler divergence (see Materials and Methods of main text). Fig. S3 shows that the distributions from *Smoldyn* become increasingly similar to the result from the well-mixed Gillespie for

increasing diffusion constants of monomer and dimer, although the high state is always underrepresented. This discrepancy can be understood and remedied as follows. Fig. S4 shows that for increasingly fast $k_{+3} = k_{-3}$ the obtained distributions from simulations resemble more and more the result from Gillespie of the conventional Schlögl model, albeit for a larger B value. We suspect this has to do with the fact that the dimerization reaction is second order while the reverse reaction is first order. Thus diffusion only affects the forward reaction by slowing it down, thus favoring the uninduced low state. This is corrected by the larger B value.

[1] Schlögl F (1972) Chemical reaction models for non-equilibrium phase transitions. Z Physik **253**: 147-161.

[2] Gaspard P (2004) Fluctuation theorem for nonequilibrium reactions. J Chem Physics **120**: 8898-8905.

[3] Hanggi P, Grabert H, Talkner P, Thomas H (1984) Bistable systems: master equation versus Fokker-Planck modelling. Phys Rev A **29**: 371-378.

[4] Vellela M, Qian H (2009) Stochastic dynamics and non-equilibrium thermodynamics of a bistable chemical system: the Schlögl model revisited. J Royal Soc Interfaces **6**: 925-940.

[5] Dykman MI, Mori E, Ross J, Hunt PM (1994) Large fluctuations and optimal paths in chemical kinetics. J Chem Phys **100**: 5735-5749.

[6] Ebeling W, Schimansky-Geier L (1979) Stochastic dynamics of a bistable reaction system. Physica **98A**: 587-600.

[7] Ge H, Qian H (2009) Thermodynamic limit of a nonequilibrium steady state: Maxwell-type construction for a bistable biochemical system. Phys Rev Lett **103**: 148103.

[8] Landauer R (1975) Inadequacy of entropy and entropy derivatives in characterizing the steady state. Phys Rev A **12**: 636-638.

[9] Andresen B, Zimmermann EC, Ross J (1984) Objections to a proposal on the rate of entropy production in systems far from equilibrium. J Chem Phys **81**: 4676-4677.

[10] Sawada Y (1981) A thermodynamic variational principle in non-equilibrium phenomena. Prog Theor Phys **66**: 68-76.

[11] Andrews SS, Addy NJ, Brent R, Arkin AP (2010) Detailed simulations of cell biology with Smoldyn 2.1. PLoS Comp Biol **6**: e1000705.
