# Supplementary figures and images for: Bistability: Requirements on Cell-Volume, Protein Diffusion, and Thermodynamics
Source: PLoS One. 2015 Apr 15;10(4):e0121681. doi: 10.1371/journal.pone.0121681 (PMC4398428; doi:10.1371/journal.pone.0121681)

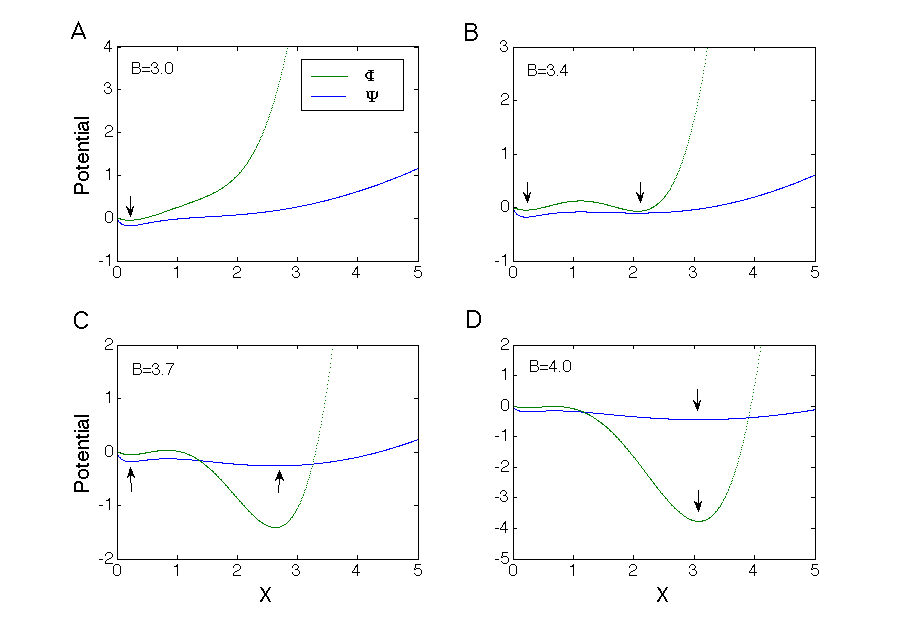

Supplement: S1 Fig — For concentration x the deterministic potentials Ψ(x) in green is calculated with Eq. 44 in S1 Text, while the stochastic potential Φ(x) in blue is calculated with main-text Equation 4 (Eq. 19 of S1 Text). (A) Arrow indicates that for B = 3.0 both potentials predict the low state as most stable. (B) Arrows indicate that around B = 3.4 the deterministic potential predicts coexistence of the low and high states. (C) Arrows indicate that around B = 3.7 the stochastic potential predicts coexistence. (D) At B = 4.0 both potentials predict the high state as most stable. (TIF) [file pone.0121681.s002.tif]

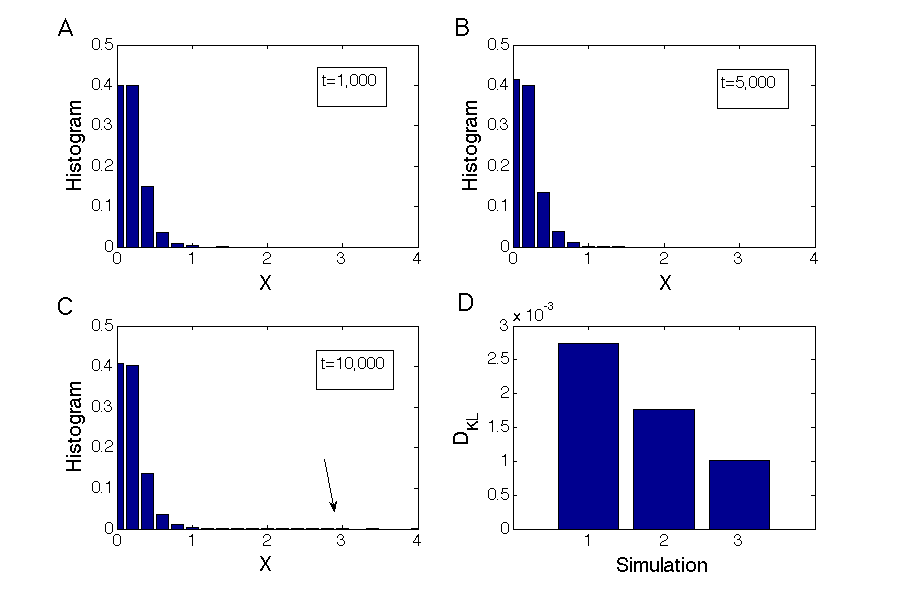

Supplement: S2 Fig — Remaining parameters were chosen as in Fig 4D with x the monomer concentration. (A-C) Simulation time is increased from t = 1,000, 5,000, to 10,000 as indicated. Arrow in (C) points to high state. (D) Kullback-Leibler divergence between each of the three simulations and Smoldyn simulation for t = 50,000 as reference distribution (see S1 Text for details). (TIF) [file pone.0121681.s003.tif]

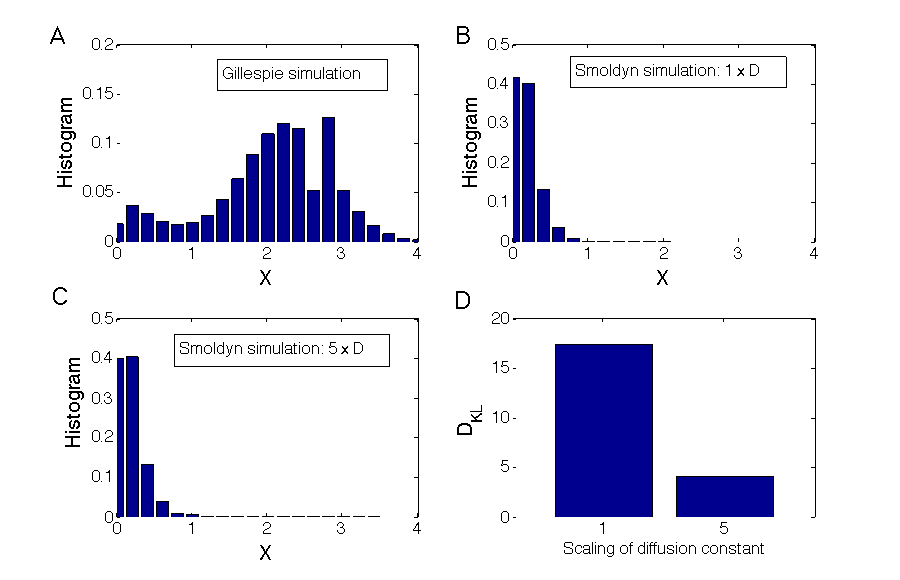

Supplement: S3 Fig — Remaining parameters were chosen as in Fig 4D with x the monomer concentration. (A) Gillespie simulation. (B) Smoldyn simulation for D = 3 (X) and 1 (X 2). (C) Smoldyn simulation for faster diffusion with D = 15 (X) and 5 (X 2). (D) Kullback-Leibler divergence between each of the two Smoldyn simulations and Gillespie simulation as reference distribution (see S1 Text for details). (TIF) [file pone.0121681.s004.tif]

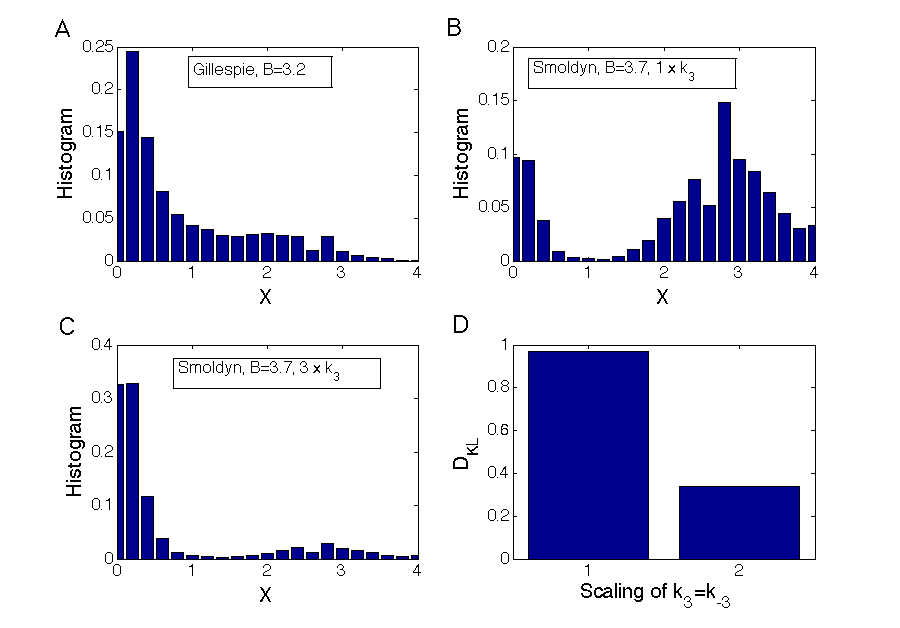

Supplement: S4 Fig — (A) Gillespie simulation for B = 3.2. (B) Smoldyn simulation for k +3 = k −3 = 1. (C) Smoldyn simulation for k +3 = k −3 = 3. (D) Kullback-Leibler divergence between each of the two Smoldyn simulations and Gillespie simulation as reference distribution (see S1 Text for details). (TIF) [file pone.0121681.s005.tif]

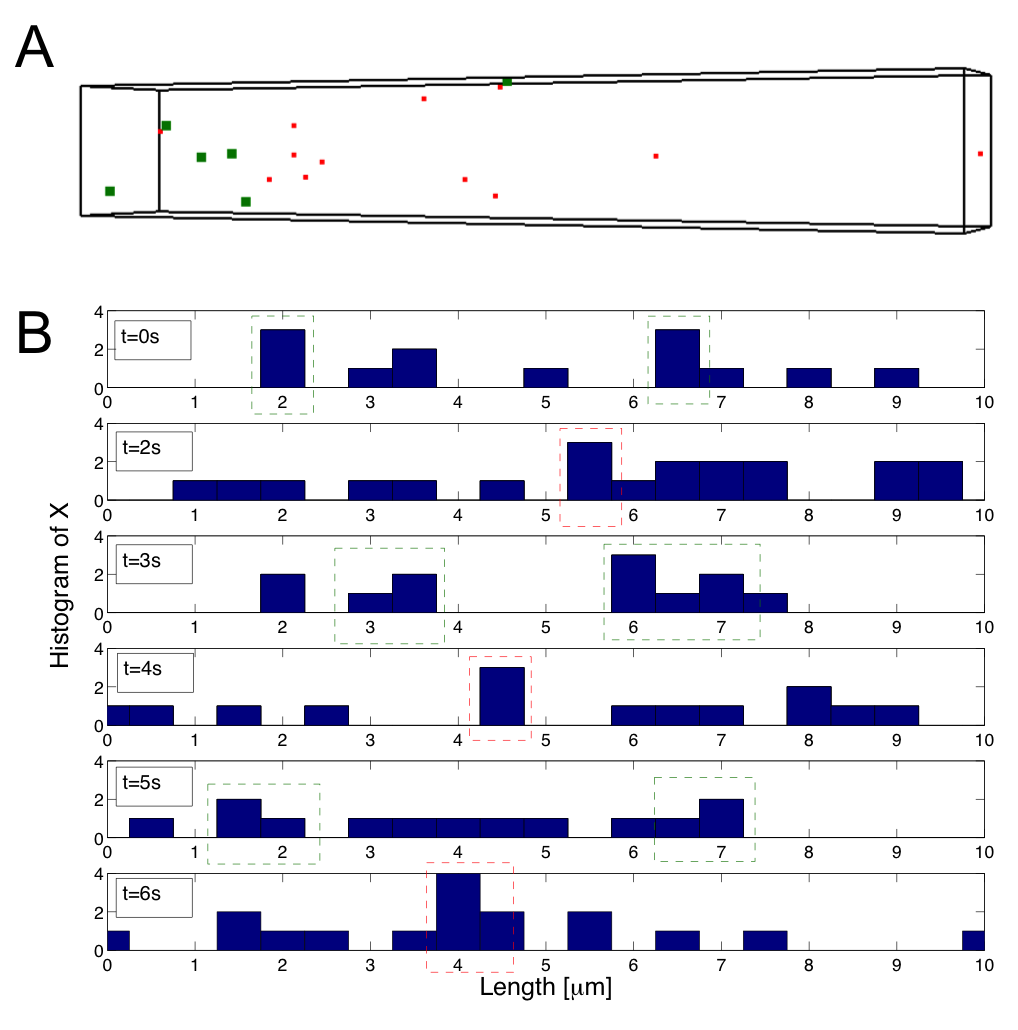

Supplement: S5 Fig — Scaling factor multiplies set of diffusion constants, D = 3 (X) and 1 (X 2). Remaining parameters as in S3B Fig. (TIF) [file pone.0121681.s006.tif]

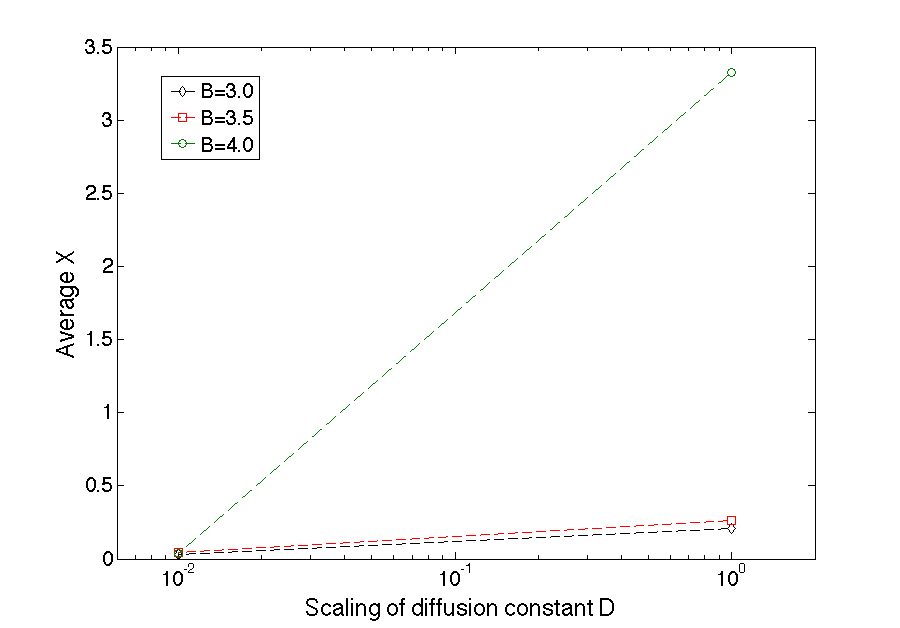

Supplement: S6 Fig — Image analysis similar to Fig 8B but with total intensity normalized by cell area to provide the intensity density. (TIF) [file pone.0121681.s007.tif]
